# Filopodome Mapping Identifies p130Cas as a Mechanosensitive Regulator of Filopodia Stability

**DOI:** 10.1016/j.cub.2018.11.053

**Published:** 2019-01-21

**Authors:** Guillaume Jacquemet, Aki Stubb, Rafael Saup, Mitro Miihkinen, Elena Kremneva, Hellyeh Hamidi, Johanna Ivaska

**Affiliations:** 1 Turku Centre for Biotechnology, University of Turku and Åbo Akademi University, Turku, Finland; 2 Institute of Biotechnology, University of Helsinki, PO Box 56, 00014 Helsinki, Finland; 3 Department of Biochemistry, University of Turku, Turku, Finland

**Keywords:** filopodia, adhesion, SIM, p130Cas, filopodome, filopodia tip adhesion, mechanotransduction, stiffness, integrin

## Abstract

Filopodia are adhesive cellular protrusions specialized in the detection of extracellular matrix (ECM)-derived cues. Although ECM engagement at focal adhesions is known to trigger the recruitment of hundreds of proteins (“adhesome”) to fine-tune cellular behavior, the components of the filopodia adhesions remain undefined. Here, we performed a structured-illumination-microscopy-based screen to map the localization of 80 target proteins, linked to cell adhesion and migration, within myosin-X-induced filopodia. We demonstrate preferential enrichment of several adhesion proteins to either filopodia tips, filopodia shafts, or shaft subdomains, suggesting divergent, spatially restricted functions for these proteins. Moreover, proteins with phosphoinositide (PI) binding sites are particularly enriched in filopodia. This, together with the strong localization of PI(3,4)P_2_ in filopodia tips, predicts critical roles for PIs in regulating filopodia ultra-structure and function. Our mapping further reveals that filopodia adhesions consist of a unique set of proteins, the filopodome, that are distinct from classical nascent adhesions, focal adhesions, and fibrillar adhesions. Using live imaging, we observe that filopodia adhesions can give rise to nascent adhesions, which, in turn, form focal adhesions. We demonstrate that p130Cas (BCAR1) is recruited to filopodia tips via its C-terminal Cas family homology domain (CCHD) and acts as a mechanosensitive regulator of filopodia stability. Finally, we demonstrate that our map based on myosin-X-induced filopodia can be translated to endogenous filopodia and fascin- and IRSp53-mediated filopodia.

## Highlights

• A super-resolution microscopy screen reveals filopodia core and accessory proteins
• FAK and paxillin are accessory, and not core, components of filopodia tip adhesions
• PI(3,4)P2 is the prominent phosphatidylinositol species enriched at filopodia tips
• p130Cas is a mechanosensitive regulator of filopodia stability

## Teaser

Jacquemet et al. perform super-resolution imaging of filopodia and define a map of core and accessory components based on a pool of target proteins linked to cell adhesion. They show that filopodia adhesions are distinct from classical adhesions and nucleate nascent adhesions and are mechanosensitive, requiring phospho-p130Cas for stabilization.

## Introduction

The ability of cells to migrate *in vivo* is necessary for many physiological processes, including embryonic development, tissue homeostasis, and wound healing. Cell migration is also implicated in distinct pathological conditions, such as inflammation and cancer metastasis. To migrate, cells interact with their environment, the extracellular matrix (ECM), via adhesion receptors, such as integrins, which provide a physical link between the ECM and the actin cytoskeleton [1]. Integrin function is controlled by a conformational switch between active and inactive states that determines ECM ligand interaction and subsequent receptor signaling [2]. Integrin activation can be triggered from within the cell by several mechanisms, including the Rap1-RIAM-talin pathway. In 2D, integrin-ligand engagement leads to the assembly of large signaling platforms, termed focal adhesions (FAs), which are composed of hundreds of proteins collectively termed the adhesome [3, 4]. FAs are highly dynamic and complex structures that develop from force-dependent maturation of nascent adhesions at the leading edge and which undergo integrin-specific centripetal translocation to form fibrillar adhesions. Importantly, FAs not only provide anchorage but also represent integrin heterodimer-ligand-specific [5] and/or ECM-ligand-specific signaling nodes with mechanosensing functions [6] and therefore constitute ideal signaling platforms for ECM recognition.

Cell motility through complex 3D microenvironments also requires efficient probing of the cell surroundings, including ECM and neighboring cells, via specialized sensory protrusions, such as filopodia [7]. Filopodia, finger-like actin-rich protrusions widely used by cells *in vivo* during normal processes (e.g., development, immune surveillance, and wound healing) and also during tumorigenesis and cancer cell dissemination [7, 8], are often the very first point of contact between a cell and its immediate surroundings. To this end, filopodia contain cell-surface receptors, such as integrins, cadherins, and growth factor receptors, that can interact with, and interpret, a wide variety of cues. In the context of ECM recognition, cues include ECM gradients [9], ECM topography [10], or ECM stiffness [11, 12]. Thus, filopodia contribute to haptotaxis [9] and possibly to durotaxis [13]. Filopodia are very dynamic structures that stabilize upon ECM tethering and formation of an adhesive structure, at their tips, termed filopodia adhesion. ECM attachment at filopodia is dependent on integrins, which are actively transported to filopodia tips by the motor protein myosin-X (MYO10) [14]. In addition, filopodia stabilization requires both talin-mediated integrin activation and integrin downstream signaling at the filopodium tip [15]. However, the composition of filopodia adhesions remains poorly defined.

The composition and architecture of FAs have been extensively studied using both microscopy and mass-spectrometric-based strategies [4, 16]. Importantly, the compilation of FA components, either by literature curation (Geiger adhesome) [3] or by proteomic approaches (consensus adhesome) [4], have led to significant advances in our understanding of adhesion-mediated processes. Considering that filopodia are significant structures *in vivo* that are also implicated in various biological processes and pathologies, such as angiogenesis and cancer progression [8], a more detailed analysis of proteins recruited to filopodia adhesions is likely to be fundamental to better understand filopodia functions. Filopodia are relatively small and labile structures (1- to 5-μm length and 50- to 200-nm width) and are therefore difficult to purify in a scale sufficient to perform mass spectrometry analyses. Here, to characterize the composition of filopodia tip adhesions, we used a targeted approach and mapped the localization of 80 proteins, implicated in cellular adhesion or protein-membrane lipid interactions, using structured illumination microscopy (SIM).

## Results

### Mapping Protein Localization in Filopodia Using SIM

**Figure 1:**
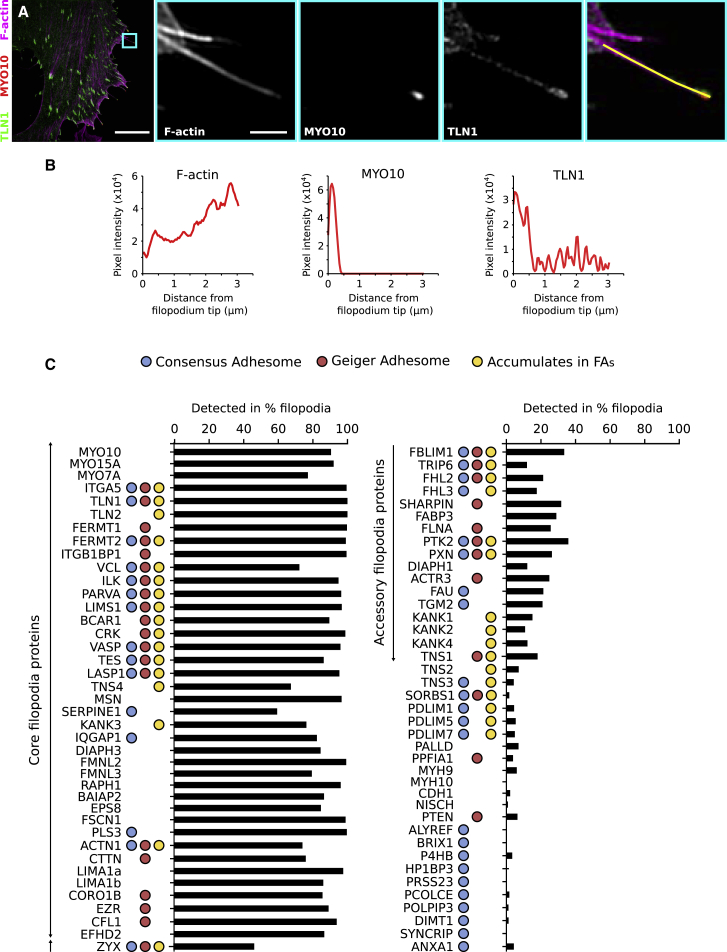
Mapping Adhesion Proteins to Filopodia Using Structured Illumination Microscopy (A–C) U2OS cells expressing a GFP/RFP-tagged protein of interest (POI) and GFP/RFP-MYO10 were plated on fibronectin for 2 hr, stained for F-actin, and imaged using structured illumination microscopy (SIM). POI distribution within filopodia (from tip to base) was assessed with line intensity profiles (n numbers can be found in Data S1). (A) An example illustrating the distribution of GFP-TLN1 (talin-1), MYO10-mScarlet (myosin-X), and F-actin within filopodia. Blue square highlights the region of interest (ROI) that is magnified; scale bars: (main) 10 μm; (inset) 1 μm; yellow line was used to measure the TLN1, MYO10, and F-actin intensity profiles. (B) Results of the line intensity profiles from (A). (C) The percentage of filopodia positive for each POI as determined from line intensity profiles. Colored circles indicate POI inclusion in the Geiger adhesome [3] (red circle) and/or the consensus adhesome [4] (blue circle) and/or accumulation in focal adhesions (FAs) (this study, yellow circle); “core filopodia proteins” and “accessory filopodia proteins” are labeled. See also Data S1 and S2.

**Figure 2:**
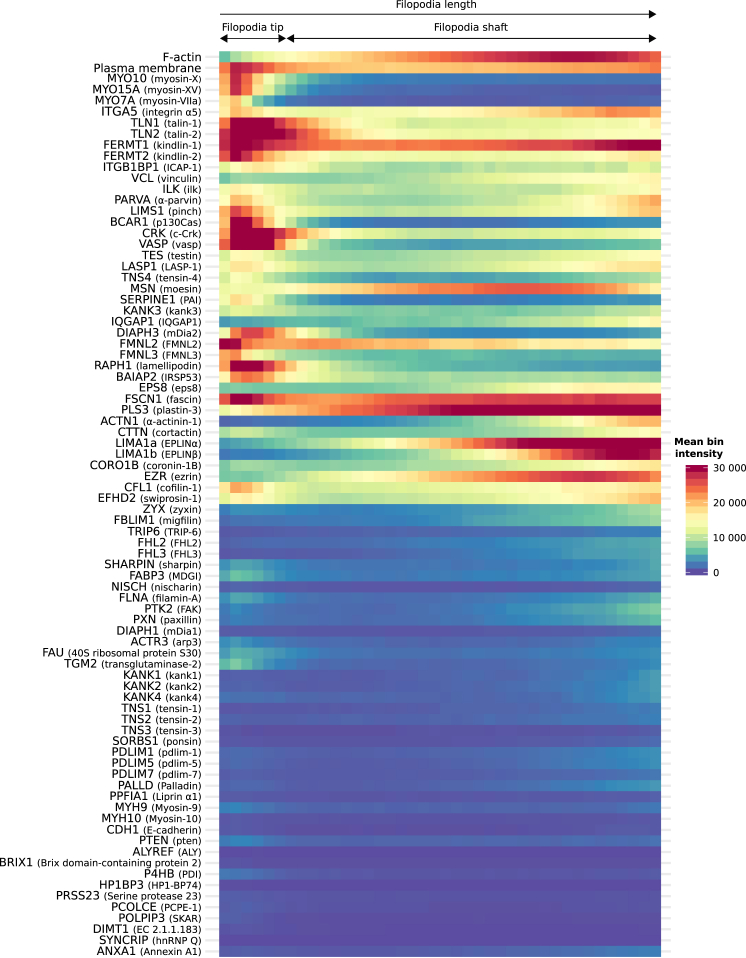
Generation of a Filopodia Map Using Correlative Imaging and Intensity Profile Averaging Heatmap demonstrating the subcellular localization of each POI within filopodia based on >250 intensity profiles (measured as in Figure 1; n numbers can be found in Data S1). POIs are labeled using their official human gene name. To create this map, intensity profiles of each POI were binned (40 bins per filopodium for each intensity profile) and then averaged (see STAR Methods for more details) and displayed as a heatmap. POIs are labeled using their official human gene name; filopodium tip (defined by MYO10) and filopodium shaft are highlighted. See also Figure S1 and Data S1 and S2.

To identify proteins that localize to filopodia, we performed a SIM-based screen, spanning 80 putative regulators of cellular adhesion or protein-membrane lipid association. Proteins of interest (POIs) (Data S1) included known filopodia components, actin regulators, established FA components (Geiger adhesome) [3], and adhesion proteins consistently identified in multiple mass spectrometry studies (consensus adhesome) [4]. We chose to visualize all the POIs as GFP-fusion proteins, as it was not feasible to generate, validate, and optimize antibodies against all endogenous POIs. Cells adhering to fibronectin and transiently co-expressing a GFP-tagged POI and red fluorescent protein (RFP)-MYO10 (to induce and visualize filopodia tips) were stained for F-actin and imaged using SIM (Figures 1A and 1B). SIM images of each POI are provided as Supplemental Information (Data S1 and S2). Strikingly, 15 of the 38 consensus adhesome proteins [4] imaged did not display clear accumulation in FAs (Figure 1C; Data S2), suggesting that they may contribute to other cell-ECM interfaces. To study POI localization and distribution along filopodia, line intensity profiles, manually drawn from filopodium tip to base (Figures 1A and 1B), were obtained for >200 filopodia per POI. Importantly, to evaluate POI distribution across multiple cells, the brightness and contrast of each image was automatically adjusted using the brightest cellular structure as the upper limit. Line intensity profiles were used to determine the percentage of filopodia positive for each POI (Figure 1C), filopodia length (Figure S1), and to create a map highlighting the distribution of the POIs within filopodia (Figure 2; see STAR Methods for details). The ImageJ macro and R scripts used to perform these quantifications are available as supplemental files (Data S2).

### Protein Mapping Reveals Classes of Core and Accessory Filopodia Proteins

Multiple established filopodia-localizing proteins, including talin-1 (TLN1) [17], formin-like protein 3 (FMNL3) [18], lamellipodin (RAPH1) [19], vasodilator-stimulated phosphoprotein (VASP) [20], mDia2 (DIAPH3) [21], and fascin (FSCN1) [22] were clearly detected with high resolution in filopodia, validating our approach. Our comprehensive mapping revealed that the proteins imaged here could be organized into three categories according to detection frequency within filopodia. The first category is composed of POIs that are primarily absent from filopodia (Data S2; Figure 1C), and due to low detection rate, we consider these proteins not to be filopodia proteins. The second category of POI is detected in a high proportion of filopodia (60%–100%), indicating that these proteins are core filopodia proteins (Data S2; Figure 1C). The third category of POI is those reliably detected but present in only a small fraction of filopodia (10%–40%), suggesting that these proteins may be accessory filopodia proteins contributing to filopodia-specific functions or defining subsets of biologically distinct filopodia (Data S2; Figure 1C).

### Phosphoinositide PI(3,4)P_2_ Is Enriched at Filopodia Tips

**Figure 3:**
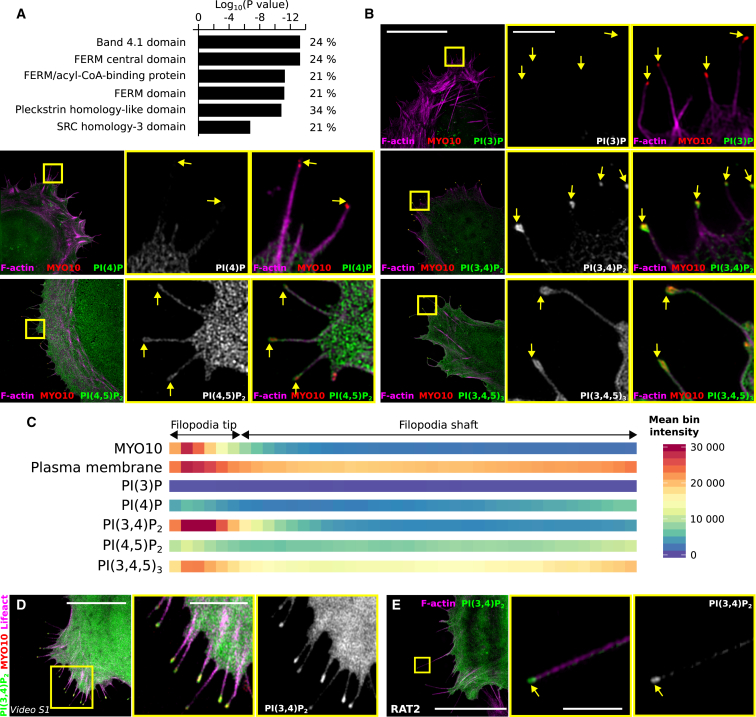
Mapping of Phosphoinositides within Filopodia (A) Functional annotation analysis (protein domain enrichments) of the core filopodia proteins (detected in ≥60% of filopodia) was performed using the INTERPRO database integrated within the Database for Annotation, Visualization, and Integrated Discovery (DAVID) platform. (B) Distribution of phosphoinositides (PIs) in filopodia. U2OS cells transiently expressing MYO10-mScarlet and GFP-tagged probes, binding with high affinity to a single PI species (PI species, probe used; PI(3)P, GFP-FYVE-PH; PI(4)P, GFP-P4M; PI(4,5)P_2_, GFP-PLC(δ1)-PH; PI(3,4,5)P_3_, GFP-BTK-PH; PI(3,4)P_2_, GFP-TAPP-PH), were plated on fibronectin for 2 hr, fixed, stained for F-actin, and imaged using SIM. Maximum intensity projections (MIPs) are displayed; scale bars: (main) 20 μm; (inset) 2 μm; yellow arrows highlight filopodia tips. (C) The distribution of each probe within filopodia was then analyzed and displayed as described in Figures 1 and 2. (D) U2OS cells expressing Lifeact-mTurquoise2, GFP-TAPP-PH, and MYO10-mScarlet were plated on fibronectin and imaged live using an Airyscan confocal microscope (1 picture every 9 s; Video S1). Scale bars: (main) 15 μm; (inset) 5 μm. (E) RAT2 cells expressing GFP-TAPP-PH were plated on fibronectin for 2 hr, stained for F-actin, and imaged using SIM. A representative MIP is displayed; scale bars: (main) 20 μm; (inset) 2 μm; yellow arrows highlight filopodia tips. See also Data S1 and S2.

To uncover common elements among the core filopodia proteins identified here, a protein domain enrichment analysis was performed (Figure 3A). This analysis revealed that proteins containing the four-point-one, ezrin, radixin, moesin (FERM) (24%) and/or SRC homology 3 (SH3) (21%) and/or pleckstrin homology (PH)-like (34%) domains are enriched in filopodia (Figure 3A). A strong enrichment of proteins containing PH-like domains led us to speculate that the phosphoinositide (PI) composition of filopodia could be a key contributor to filopodia function.

To map the distribution of the various PI in filopodia, GFP-tagged probes with high affinity to a single PI species [23] were imaged using SIM and analyzed as described above (Figures 3B and 3C). PI(3)P (imaged using GFP-FYVE-PH) was mostly detected on vesicular structures within the cell body, but not in filopodia (Figures 3B and 3C). PI(4)P (imaged using GFP-P4M) localized at the plasma membrane and on intracellular vesicles but was only weakly detected within filopodia (Figures 3B and 3C). In contrast, PI(4,5)P_2_ (labeled with GFP-PLCγPH) and PI(3,4,5)P_3_ (labeled with GFP-BTK-PH) were both strongly detected within filopodia with a relatively homogeneous distribution (similar to the plasma membrane). Strikingly, PI(3,4)P_2_ (labeled using GFP-TAPP-PH) was also detected in filopodia, but in contrast to the other PI, PI(3,4)P_2_ was strongly enriched to filopodia tips. Importantly, PI(3,4)P_2_ accumulation at filopodia tips was confirmed in live cells (Figure 3D; Video S1) and in endogenous filopodia (Figure 3E). This accumulation of PI(3,4)P_2_ to filopodia tips is surprising and suggests that PI(3,4)P_2_ contributes to filopodia tip functions or organization. Future work will aim at identifying the role of PI(3,4)P_2_ in filopodia as well as the proteins that are responsible for its accumulation at filopodia tips.

Video S1. PI(3,4)P_2_ Dynamics in Filopodia, Related to Figure 3 U2OS cells expressing MYO10-mScarlet, GFP-TAPP-PH and lifeact-mTurquoise2 were plated on fibronectin for 2 h before being imaged live using an Airyscan confocal microscope. The yellow square highlights a ROI, which is magnified.

### SIM Mapping Reveals Novel Filopodia Tip Proteins and Subdomains within Filopodia Shafts

**Figure 4:**
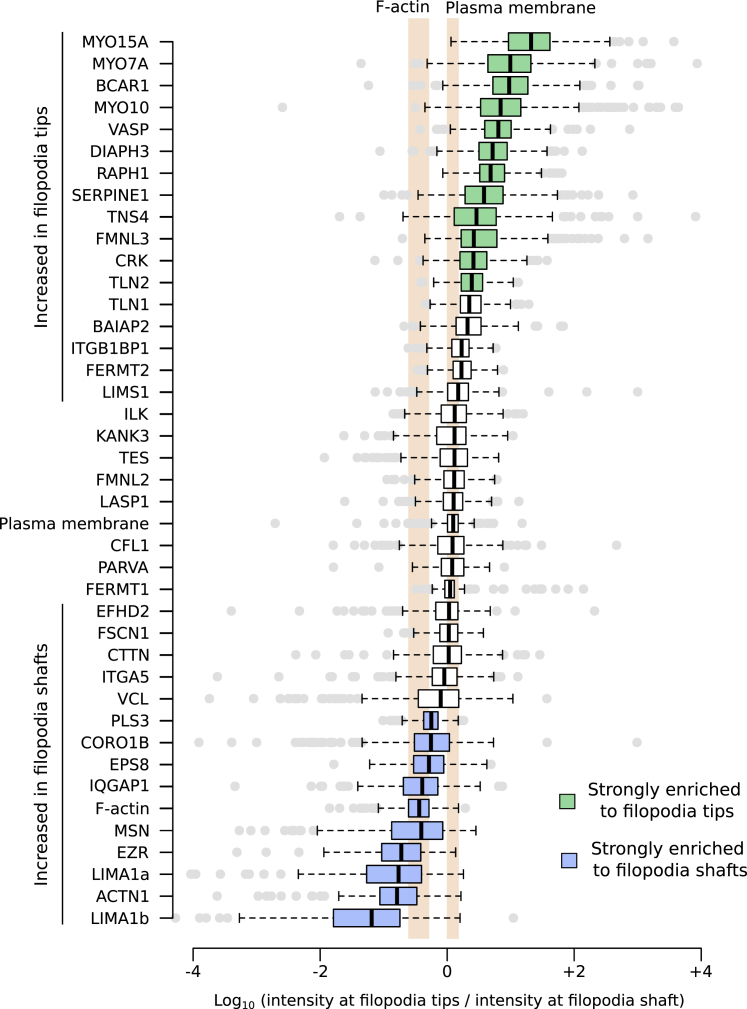
Preferential Enrichment of Adhesion Proteins to Filopodium Shaft or Tip The preferential recruitment of core filopodia proteins to filopodia tips or shafts was assessed by calculating an enrichment ratio (averaged intensity at filopodium tip versus shaft). Results are displayed as Tukey boxplots using a logarithmic scale, and the POIs are ordered as a function of the median enrichment score. Enrichment scores of F-actin and the plasma membrane (labeled by CAAX-GFP) are highlighted in orange. A median enrichment score displaying ≥two-fold change over that of the plasma membrane was considered to represent strong POI enrichment to either filopodia tips (≥two-fold increase, highlighted in green) or to filopodia shafts (≥two-fold decrease, highlighted in blue). Statistically significant enrichment scores are noted as either “increased in filopodia tips” or “increased in filopodia shafts.” See also Data S1 and S2.

To quantitatively analyze the preferential recruitment of the core filopodia proteins, identified here, to filopodia tips or shafts, an enrichment ratio was calculated (Figure 4). As expected, proteins known to compose the tip complex, such as MYO10 [14], VASP [20], DIAPH3 [21], FMNL3 [18], and RAPH1 [19], were strongly enriched to filopodia tips (Figure 4). Importantly, our analysis revealed that F-actin is low in the filopodium tip compared to the filopodium shaft. Conversely, the amount of plasma membrane was slightly enriched at filopodium tips compared to shafts (Figures 2 and 4). This is likely due to the formation of a bud at the end of filopodia. Therefore, the enrichment score of a POI was compared to the enrichment score obtained for the plasma membrane rather than the enrichment score of F-actin (Figure 4).

Our mapping revealed several original filopodia tip proteins, such as p130Cas (BCAR1), tensin-4 (TNS4), and crk (CRK). Other proteins displaying preferential recruitment to filopodia tips over filopodia shafts include the integrin activity modulators TLN1 and talin-2 (TLN2), kindlin-2 (FERMT2), and ICAP-1 (ITGB1BP1; Figures 2 and 4). Proteins strongly enriched to filopodia shafts include predominantly actin-regulating and cross-linking proteins, such as eplins (LIMA1a and LIMA1b), alpha-actinin-1 (ACTN1), plastin (PLS3), and ezrin (EZR) (Figures 2 and 4). Interestingly, eps8 (EPS8) and IRSp53 (BAIAP2), two regulators of filopodia formation, which are also known to interact with each other [24], appear to be spatially segregated within established filopodia as BAIAP2 accumulates at filopodia tips while EPS8 is enriched to filopodia shafts (Figures 2 and 4). Filopodia shafts can be segmented into subdomains depending on the degree of penetration of certain proteins throughout the filopodium (from the base of the shaft toward the tip). For instance, ACTN1 labels only the base (first 20%) of the shaft and proteins, such as EZR or LIMA1, label the first 50%–60% portion of the shaft (Figure 2). The segregation of the filopodia shaft into subdomains could indicate that these segments are also functionally different.

### Filopodia Adhesions Are Distinct from Other Adhesions

In migrating cells, adhesion sites are highly dynamic structures that undergo a well-defined force-dependent maturation sequence from nascent adhesions to fibrillar adhesions [25]. Our SIM-based mapping allowed us to identify several established integrin binders and integrin activity regulators (TLN1 and TLN2; FERMT1 and FERMT2; and ITGB1BP1) as core filopodia proteins, while other integrin binders and regulators, such as tensin-1 (TNS1), tensin-3 (TNS3), MDGI (FABP3), filamin-a (FLNA), and nischarin (NISCH), were absent from filopodia. In addition, components of the signaling modules BCAR1-CRK and the Ilk-pinch-parvin complex (ILK, LIMS1, and PARVA; IPP complex) were detected in filopodia with similar distributions (Figures 1 and 2). Several proteins accumulating in FAs were also detected in most filopodia, including VASP, ACTN1, testin (TES), LASP-1 (LASP1), and vinculin (VCL).

Filopodia are regions of low force in the cell [7], and therefore, it is not surprising that proteins associated with mature and/or fibrillar adhesions were not identified as core filopodia proteins. These proteins include the actin-binding tensin isoforms (TNS1–3) [26], PDLIM1/5/7 [27], TRIP6 [27], zyxin (ZYX) [26], and palladin (PALLD) [28] (Figures 1 and 2). In addition, the major non-muscle myosins mediating cellular contractility, non-muscle myosin heavy chain IIa (MYH9) and non-muscle myosin heavy chain IIb (MYH10), were not detected in filopodia. Importantly, multiple proteins associated with the low force-bearing nascent adhesions, including paxillin (PXN) [29], FAK (PTK2) [30], or arp3 (ACTR3) [31], were classified as filopodia accessory, not core, proteins, as they were detected in <40% of filopodia (Figures 1 and 2). Thus, our SIM mapping indicates that filopodia adhesions consist of a unique set of proteins, the filopodome, and are distinct from classical nascent adhesions, FAs and fibrillar adhesions.

### Filopodia Adhesions Nucleate Nascent Adhesions

**Figure 5:**
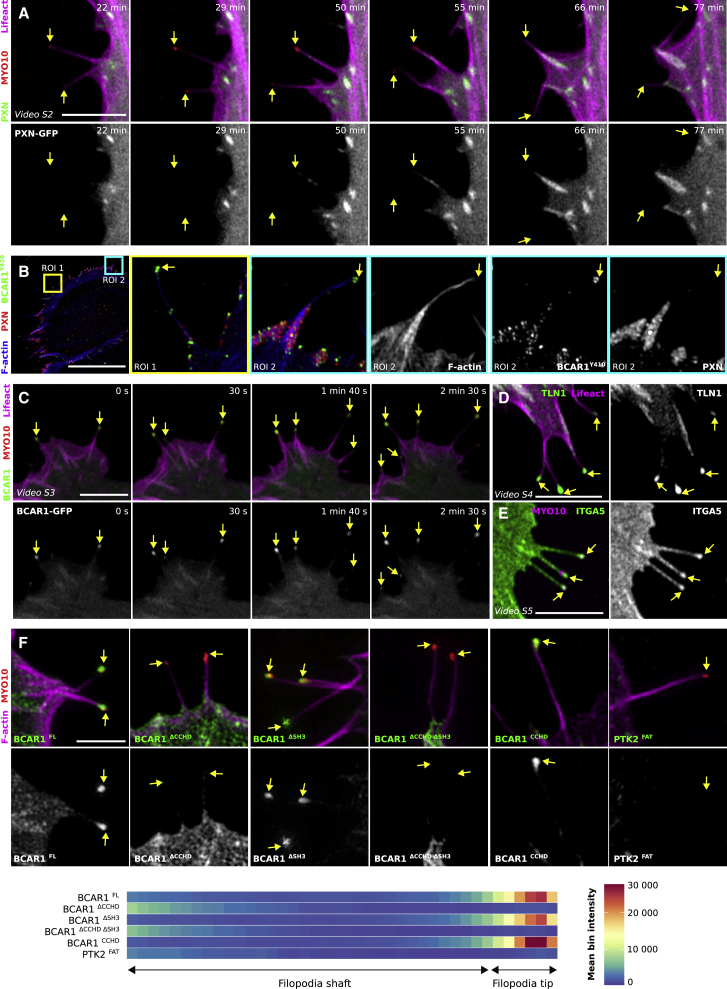
Filopodia Tip Adhesions (A) U2OS cells expressing Lifeact-mTurquoise2, PXN-mEmerald, and MYO10-mScarlet were plated on fibronectin and imaged live using an Airyscan confocal microscope (1 picture every 30 s; scale bar, 5 μm; Video S2). Images highlight time points of interest in a magnified area; yellow arrows highlight filopodia tips. (B) U2OS cells expressing MYO10-mScarlet were plated on fibronectin for 2 hr; stained for F-actin, phospho-BCAR1 (Y410), and endogenous PXN; and imaged using SIM. A representative MIP is displayed. The blue and yellow squares highlight ROIs, which are magnified; scale bars: (main) 20 μm; (inset) 2 μm. (C–E) Live-cell imaging of U2OS cells transiently expressing MYO10-mScarlet together with either Lifeact-mTurquoise2 and BCAR1-eGFP (C; Video S3), Lifeact-mTurquoise2 and TLN1-eGFP (D; Video S4), or ITGA5-GFP (E; Video S5). Cells were plated on fibronectin and imaged live using an Airyscan confocal microscope. Yellow arrows highlight filopodia tips; scale bars: 5 μm. (F) U2OS cells expressing MYO10-mScarlet together with either GFP-BCAR1 full-length (FL), BCAR1 deletion constructs, or GFP-PTK2-FAT (PTK2^FAT^) were plated on fibronectin for 2 hr, stained for F-actin, and imaged using SIM. BCAR1^ΔSH3^, BCAR1 SH3 domain deletion; BCAR1^ΔCCHD^, BCAR1 CCHD domain deletion; BCAR1^ΔCCHDΔSH3^, BCAR1 SH3 domain and CCHD deletion; BCAR1^CCHD^, BCAR1 CCHD domain alone. Images highlight magnified areas of interest; scale bar: 2 μm; full fields of view are available in Figure S4. Distribution of each construct within filopodia was analyzed and displayed as described in Figures 1 and 2. See also Figures S2, S3, and S4.

The absence of PXN or PTK2 in >60% of filopodia was unexpected, as there are documented interactions between these two proteins and a large number of the core filopodia proteins identified here (Figure S2A). In line with our mapping data using GFP-tagged proteins, we found that endogenous ILK and TLN1/2 localize to filopodia and PXN and PTK2 were observed only in a small percentage of filopodia (Figures S2B–S2E). When PXN was detected within filopodia, its localization varied between being detected at the tip and/or in the shaft (Figure S2E), and we hypothesized that the variable localization of PXN in filopodia could be linked to filopodia dynamics. Previous work has reported MYO10 localization in nascent adhesion prior to filopodia elongation [32], but we did not observe this phenomenon here. Live-cell imaging revealed that unstable filopodia are devoid of PXN (Figure 5A; Video S2), whereas, in stable filopodia, PXN is initially detected briefly at the tip, followed by localization to, and increased clustering in, filopodia shafts, possibly due to actin retrograde flow. Upon lamellipodia advancement, these clusters of PXN gave rise to FAs (Figure 5A; Video S2). These data suggest that stabilized filopodia adhesions can trigger the nucleation of nascent adhesions.

Video S2. Paxillin Dynamics in Filopodia, Related to Figure 5 U2OS cells expressing MYO10-mScarlet, PXN-GFP and lifeact-mTurquoise2 were plated on fibronectin for 2 h before being imaged live using an Airyscan confocal microscope. The white square highlights a ROI, which is magnified.

### BCAR1 Is a Novel Component of the Filopodia Tip Complex

Talin-mediated integrin activation and linkage to the actin cytoskeleton are required for filopodia stabilization and maturation into adhesions [15]. As adhesion maturation strongly correlates with increasing forces exerted on the substrate, this prompted us to review our core filopodia proteins for other potential force sensors. BCAR1 (p130Cas), one of our filopodia core components (Figure 1) strongly enriched in filopodia tips (Figure 4), has been implicated as a mechanosensitive protein that becomes phosphorylated by SRC in response to mechanical stretch [33]. We first validated our mapping data by staining endogenous BCAR1 and found that it indeed localizes to filopodia tips (Figure S3A). In addition, a phosphorylation-specific antibody indicated that endogenous BCAR1 is phosphorylated at filopodia tips, where it co-localizes with MYO10, but not with PXN (Figures 5B and S3B). Live-cell imaging also confirmed our mapping data, as BCAR1 was always observed at filopodia tips, regardless of filopodia stability (Figure 5C; Video S3), in a similar fashion to TLN1 (Figure 5D; Video S4), and ITGA5 distribution was relatively homogeneous (Figure 5E; Video S5). Altogether, our data indicate that BCAR1 is a constitutive component of the filopodia tip complex (Figures 1 and 5), and its phosphorylation status suggests that it may play a role in ECM sensing in filopodia adhesions.

Video S3. BCAR1 Dynamics in Filopodia, Related to Figure 5 U2OS cells expressing MYO10-mScarlet, BCAR1-GFP and lifeact-mTurquoise2 were plated on fibronectin for 2 h before being imaged live using an Airyscan confocal microscope.

Video S4. TLN1 Dynamics in Filopodia, Related to Figure 5 U2OS cells expressing MYO10-mScarlet, TLN1-GFP and lifeact-mTurquoise2 were plated on fibronectin for 2 h before being imaged live using an Airyscan confocal microscope.

Video S5. ITGA5 Dynamics in Filopodia, Related to Figure 5 U2OS cells expressing MYO10-mScarlet and ITGA5-GFP were plated on fibronectin for 2 h before being imaged live using an Airyscan confocal microscope.

### BCAR1 Is Recruited to Filopodia Tips via Its CCHD

BCAR1 localization to FAs has been suggested to be regulated via direct interaction with PXN or PTK2 [34, 35], both of which are absent from most filopodia. In addition, BCAR1 recruitment to filopodia tips is insensitive to inhibition of SRC, PTK2, or cellular contractility (Figure S3C). Together, this suggested that BCAR1 localization to filopodia tips occurs via a distinct mechanism to its FA targeting. BCAR1 is composed of five principal domains, including an N-terminal SH3 domain followed by a proline-rich region, a substrate domain, a serine-rich region, and a C-terminal Cas family homology domain (CCHD) [36]. We analyzed the subcellular location of BCAR1 constructs lacking the SH3 domain and/or the CCHD and, as previously described, observed that constructs lacking the SH3 domain or the CCHD accumulated poorly to FAs, and the construct lacking both domains was completely absent from FAs (Figure S4) [37]. Importantly, constructs lacking the CCHD failed to accumulate to filopodia tips, and the construct lacking the SH3 domain accumulated to filopodia tips to the same extent as the full-length wild-type construct (Figures 5F and S4). This indicates that BCAR1 CCHD, but not the SH3 domain, is required to target BCAR1 to filopodia tips. Importantly, BCAR1 CCHD alone is sufficient for filopodia tip localization, demonstrating that BCAR1 CCHD is both a FA-targeting domain and a filopodia-tip-complex-targeting domain (Figure 5F).

### BCAR1 Contributes to Filopodia Stabilization and Stiffness Sensing

**Figure 6:**
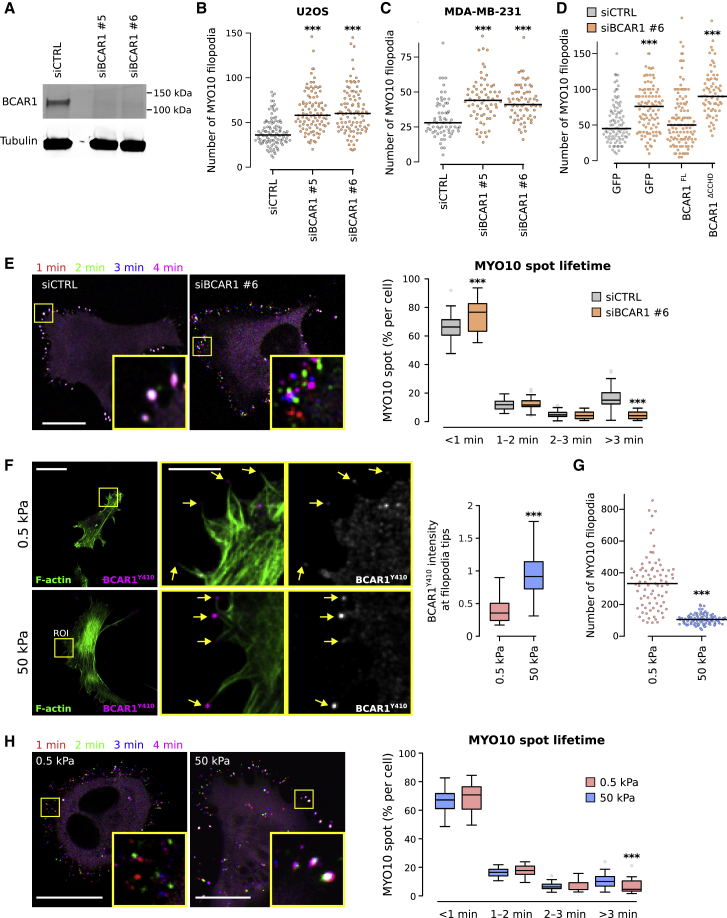
BCAR1 Regulates Environment Sensing at Filopodia Tips (A) Efficiency of siRNA-mediated (oligos nos. 5 and 6) silencing of BCAR1 in U2OS cells. (B and C) BCAR1-silenced (oligos nos. 5 and 6) U2OS (B) and MDA-MB-231 (C) cells transiently expressing MYO10-GFP were plated on fibronectin for 2 hr, fixed, and the number of MYO10-positive filopodia per cell was quantified (n > 65 cells, three biological repeats; ^∗∗∗^p value < 5.4 × 10^−6^). (D) BCAR1-silenced (oligo no. 6) U2OS cells transiently expressing MYO10-mScarlet together with GFP, GFP-BCAR1, or GFP-BCAR1^ΔCCHD^ were plated on fibronectin for 2 hr, fixed, and the number of MYO10-positive filopodia per cell was quantified (n > 69 cells, three biological repeats; ^∗∗∗^p value < 1.1 × 10^−5^). (E) BCAR1-silenced (oligo no. 6) U2OS cells transiently expressing MYO10-GFP were plated on fibronectin and imaged live using an Airyscan confocal microscope (1 picture every 5 s; scale bar, 20 μm). Representative images at different time points are shown. For each condition, MYO10-positive particles were automatically tracked, and MYO10 spot lifetime (calculated as a percentage of the total number of filopodia generated per cell) was plotted and displayed as Tukey boxplots (see STAR Methods for details; three biological repeats, more than 40 cells per condition, ^∗∗∗^p value < 8.78 × 10^−5^). (F–H) U2OS cells expressing MYO10-mScarlet (F) or MYO10-GFP (G and H) were plated on fibronectin-coated polyacrylamide gels of defined stiffness (0.5 kPa, soft; 50 kPa, stiff) for 2 hr. (F) Cells were stained for F-actin and phospho-BCAR1 (Y410) and imaged using a spinning disk confocal microscope. MIPs are displayed; yellow squares highlight ROI, which are magnified; yellow arrows highlight filopodia tips; scale bars: (main) 20 μm; (inset) 5 μm. (G) Cells were stained for F-actin, imaged using an Airyscan confocal microscope, and the number of MYO10-positive filopodia per cell was quantified (n > 81 cells, three biological repeats; ^∗∗∗^p value < 5.5 × 10^−20^). MIPs are displayed in Figure S5C. (H) Live-cell imaging on an Airyscan confocal microscope (scale bars: 20 μm). Representative images at different time points are shown. For each condition, MYO10 spot lifetime was analyzed as in (E) (three biological repeats, more than 34 cells per condition; ^∗∗∗^p value < 9.4 × 10^−4^). See also Figure S5 and Data S1 and S2.

Previously, we revealed that integrin activation as well as integrin downstream signaling are required for efficient filopodia formation and stabilization [15]. In order to investigate the potential role of BCAR1 in these processes, we silenced BCAR1 expression using two independent small interfering RNAs (siRNAs) (Figure 6A). In U2OS and MDA-MB-231 cells, silencing of BCAR1 led to an increase in the number of MYO10 filopodia (Figures 6B and 6C), and overall filopodia length remained unaffected (Figure S5A). Importantly, the effect of BCAR1 silencing on filopodia numbers could be rescued by expressing murine GFP-BCAR1, but not by expressing GFP-BCAR1 lacking the CCHD (Figure 6D). These data indicate that BCAR1, unlike TLN1 [15], is not essential for filopodia formation. However, BCAR1 is critical for efficient filopodia stabilization, as BCAR1-silenced cells displayed a higher proportion of unstable filopodia and a lower proportion of stable filopodia compared with control cells (Figure 6E).

BCAR1 silencing did not appear to impair the ability of U2OS cells to generate traction forces (Figure S5B). As BCAR1 Y410 phosphorylation is increased upon mechanical stretch, a process that enables cells to sense the mechanical properties of the ECM [33], and as BCAR1 is phosphorylated at filopodia tips (Figure 5B), we speculated that BCAR1 phosphorylation could be one of the signals, used at filopodia tips, to sense stiffness. Using fibronectin-coated polyacrylamide gels of defined stiffness (0.5 kPa, soft; 50 kPa, stiff), we found that BCAR1 Y410 phosphorylation was significantly higher at filopodia tips when cells were plated on a stiffer substrate compared to a softer environment (Figure 6F). Interestingly, when plated on a soft substrate, cells formed a much higher number of MYO10 filopodia (Figures 6G and S5C), most of which were unstable (Figure 6H). These data mirror the results obtained when BCAR1 is downregulated and cells are plated on stiff substrate (Figures 6B-6E). Taken together, our data clearly demonstrate that BCAR1 is part of the filopodia tip mechanosensitive machinery, which attenuates initiation of new filopodia downstream of filopodia stabilization.

### Taking the Filopodome beyond MYO10-Induced Filopodia

**Figure 7:**
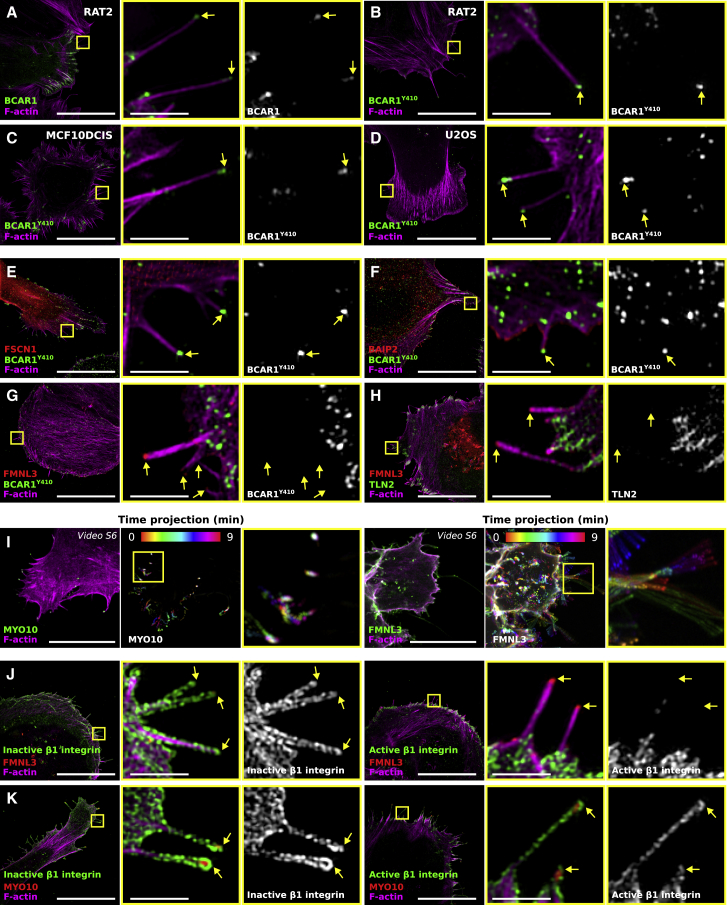
ECM Sensing in Distinct Filopodia Types (A) RAT2 cells expressing BCAR1-GFP were plated on fibronectin for 2 hr, and their endogenous filopodia were stained for F-actin and imaged using SIM. A representative MIP is displayed. (B–D) Cells were plated on fibronectin (B, RAT2 cells, 2 hr; C, MCF10DCIS.com, 2 hr; D, U2OS, 20 min) fixed, stained for F-actin and phospho-BCAR1 (Y410), and endogenous filopodia imaged using SIM. Representative MIPs are displayed. (E–G) U2OS cells expressing GFP-FSCN1 (E), GFP-BAIP2 (F), or GFP-FMNL3 (G; all known filopodia-inducing proteins) were plated on fibronectin for 2 hr, stained for F-actin and phospho-BCAR1 (Y410), and imaged using SIM. Representative MIPs are displayed. (H) U2OS cells expressing GFP-FMNL3 and mCherry-TLN2 were plated on fibronectin for 2 hr, stained for F-actin, and imaged using SIM. A representative MIP is displayed. (I) Live-cell imaging of U2OS cells transiently expressing Lifeact-mTurquoise2 and MYO10-GFP or Lifeact-mTurquoise2 and FMNL3-GFP (Video S6). Cells plated on fibronectin were imaged live using an Airyscan confocal microscope. Images display a time point of interest as well as color-coded time projections. Scale bars: 20 μm. (J and K) U2OS cells expressing GFP-FMNL3 (J) or GFP-MYO10 (K) were plated on fibronectin for 2 hr, fixed and stained for F-actin together with inactive (clone 4b4) or active (clone 12G10) β1 integrin, and imaged using SIM. MIPs are displayed. For all panels, the yellow squares highlight ROIs, which are magnified; yellow arrows highlight filopodia tips; scale bars: (main) 20 μm; (inset) 2 μm. See also Figure S6.

Here, we used MYO10-induced filopodia as a model to characterize the composition of filopodia tip adhesions (filopodome). Importantly, we could validate our key findings in endogenous filopodia, including the presence of the filopodome components RAPH1, BCAR1, VASP, and TLN1 (Figures 7A and S6A) and the absence of PXN (Figure S6A). BCAR1 Y410 phosphorylation could also be detected at the tips of endogenous filopodia in RAT2, MCF10DCIS.com and U2OS cells (Figures 7B–7D) as well as at the tip of filopodia induced by FSCN1 or BAIP2 (Figures 7E and 7F). Taken together, these data clearly demonstrate that our mapping data can be translated to other filopodia subtypes.

Interestingly, we could not detect BCAR1 Y410 phosphorylation or TLN2 at the tips of FMNL3-induced filopodia (Figures 7G and 7H), which prompted us to further analyze their composition and dynamics. Live-cell imaging revealed that FMNL3-induced filopodia hover over the substrate but fail to stabilize (Figure 7I; Video S6). In line with the lack of TLN2, the integrin pool within FMNL3-induced filopodia appears to be mostly inactive compared to the integrin activity levels in MYO10-induced filopodia (Figures 7J and 7K). Absent or reduced integrin activation in FMNL3-induced filopodia likely explains the failure of these filopodia to stabilize. These data clearly demonstrate the importance of integrin inside-out signaling in activating integrins at filopodia tips, and future work will aim at deciphering the contribution of the filopodome components in regulating integrin activity.

Video S6. Dynamics of MYO10- and FMNL3-Induced Filopodia, Related to Figure 7 U2OS cells expressing lifeact-mTurquoise2 and MYO10-GFP or lifeact-mTurquoise2 and FMNL3-GFP were plated on fibronectin and imaged live using an Airyscan confocal microscope.

## Discussion

Filopodia are sensory protrusions specialized in probing the cellular environment, including neighboring cells, chemokines, and the ECM. Here, to gain insight into the composition and biological function of filopodia tip adhesions, we mapped the sub-filopodia localization of 80 proteins linked to cell adhesion and migration using MYO10-induced filopodia as a model system (Figures 1, 2, and 4). Additionally, we validated our data in both endogenous filopodia and in filopodia induced by other factors (Figure 7); however, our map is unlikely to be applicable to all types of filopodia or filopodia-like protrusions. For instance, we report that FMNL3-induced filopodia do not form adhesions at their tips and therefore are substantially different from MYO10-induced filopodia (Figure 7). In addition, it is likely that the composition of filopodia tip adhesions described here will vary in function of the cell types and/or conditions used. For instance, as demonstrated for the adhesome [38], the ECM (biochemical composition and/or stiffness) engaged at filopodia tips is likely to tune the composition of the filopodome.

Our mapping revealed an enrichment of proteins binding to PI in filopodia (Figure 3). Mapping of multiple PI species identified spatial segregation of these PI within filopodia. PI(4,5)P_2_ and PI(3,4,5)P_3_ display a homogeneous distribution, whereas PI(3,4)P_2_ exhibits strong enrichment to filopodia tips (Figure 3). PI(4,5)P_2_ is an activator of both CDC42 and N-WASP, two promotors of filopodia formation [39], and PI(4,5)P_2_-rich lipid bilayers induce the elongation of filopodia-like structures *in vitro* [40]. PI(3,4,5)P_3_ can also induce filopodia by activating MYO10, and preventing PI(3,4,5)P_3_ generation by inhibiting phosphoinositide 3-kinase is sufficient to block filopodia formation [41]. The filopodia-tip localization of PI(3,4)P_2_ is an original observation, and its role in filopodia remains to be investigated. PI(3,4)P_2_ was reported to promote stability and maturation of invadopodia via the recruitment of Tks5 [42, 43]. Enrichment of PI(3,4)P_2_ to filopodia tips is likely to promote the accumulation of specific proteins. In our study, we found that lamellipodin (RAPH1), a known PI(3,4)P_2_ binder [19], accumulates to filopodia tips (Figures 2 and 4). *In vitro*, PI(3,4)P_2_ inhibits PI(4,5)P_2_-mediated actin polymerization [44], and therefore, PI(3,4)P_2_ could also play a role in constraining filopodia extension. Future work will aim at identifying the role of PI(3,4)P_2_ in filopodia as well as the proteins that are responsible for its accumulation in filopodia tips.

Integrin function is controlled by a conformational switch between active and inactive states that dictate integrin-ECM ligand interaction [2]. Here, we report that integrin activation, from within the cell, at filopodia, is key for filopodia stabilization and ECM sensing (Figure 7). Our mapping revealed a specific subset of known integrin binders and activity modulators that are recruited to filopodia. These include the integrin activators talins and kindlins as well as the integrin inactivators ICAP-1 (ITGB1BP1) and moesin (MSN) (Figure 2), all of which bind to the β1 integrin cytoplasmic tail on overlapping sites [45]. Future challenges will be to establish how integrin functions, including transport and activation, are coordinated by multiple adaptors within filopodia and what is the sequence and hierarchy of protein binding to the short cytoplasmic domains of integrin β subunits.

Filopodia sense ECM topography and/or ECM stiffness [10, 11, 12]. We previously observed that filopodia stabilization precedes FA maturation and that this process directs cell migration [15]. Here, we observed that PXN-positive adhesions could form in filopodia shafts, which, upon lamellipodia advancement [46], lead to the formation of FAs (Figure 5). As filopodia are widely used by cells migrating in fibrillar matrices [47, 48], it is tempting to speculate that the formation of PXN-positive adhesions in the filopodia shaft could be a mechanism by which cells sense matrix alignment. In the context of stiffness sensing, filopodia tip proteins TLN1 and BCAR1 have both previously been reported to be mechanosensitive [36]. Forces exerted by individual filopodia typically range from 5 to 25 pN (with a maximum potential of up to 2 nN) and are principally mediated by actin retrograde flow [49, 50, 51] and, upon maturation, by myosin contractility [52]. Accordingly, neither MYH9 nor MYH10 were detected within filopodia (Figures 1 and 2). In the case of TLN1, forces above 5 pN (15 pN for further activation) can induce conformational changes that trigger a switch from talin-RIAM to talin-vinculin complexes, leading to FA stabilization [53]. In the case of BCAR1, forces induce the stretching of the BCAR1 substrate domain, leading to its phosphorylation by SRC [33]. BCAR1 phosphorylation is not mediated by actomyosin contractility but instead depends on an intact actin cytoskeleton [54]. Importantly, here, we demonstrate that BCAR1 phosphorylation at filopodia tips is stiffness sensitive (Figure 6F). Although the exact amount of force required to activate BCAR1 remains unknown, the BCAR1 substrate domain is disorganized, and it is likely that the weak forces mediated by actin retrograde flow alone (5 pN in magnitude) are sufficient to mediate activation [54]. Due to the low force requirement for BCAR1 activation compared to TLN1, it is likely that, at the filopodia tip, BCAR1 will be one of the first mechanosensitive proteins to be activated. BCAR1 activation may then lead to activation of Rap1 [55, 56], which would, in turn, promote talin-mediated integrin activation, adhesion re-enforcement, and filopodia stabilization. Importantly, BCAR1-dependent filopodia stabilization functions as a feedback inhibitor for initiation of new filopodia nucleation.

Altogether, we have revealed that filopodia adhesions consist of a unique set of proteins, the filopodome, that are distinct from classical nascent adhesions, FAs, and fibrillar adhesions. Our mapping will be a valuable resource for future studies aimed at unravelling the biological relevance of filopodia in different developmental and pathological conditions involving cell motility and cellular responsiveness to environmental cues.

## STAR★Methods

### Key Resources Table

**Table:** 

REAGENT or RESOURCE	SOURCE	IDENTIFIER
**Antibodies**		

Mouse anti-human active β1 integrin 12G10 (1:100 for IF)	Abcam	catalog number: ab30394; RRID:AB_775726
Mouse anti-human inactive β1 integrin monoclonal 4B4 (1:200 for IF)	Beckman Coulter	catalog number: 6603113; RRID:AB_10638675
Mouse monoclonal anti-p130Cas (BCAR1, 1:100 for IF, 1:1000 for WB)	Santa Cruz Biotechnology	catalog number: SC-20029; RRID:AB_628064
Mouse monoclonal anti-talin-1 (TLN1, clone 8d4, 1:100 for IF)	Sigma	catalog number: T3287; RRID:AB_477572
Mouse monoclonal anti-FAK (PTK2, Clone 77, 1:100 for IF)	BD Biosciences	catalog number: 610087; RRID:AB_397494
Mouse monoclonal anti-α-tubulin (clone 12G10, 1:1000 for WB)	The Developmental Studies Hybridoma Bank	catalog number: 12G10; AB_1157911
Mouse monoclonal anti-paxillin (PXN, Clone 349, 1:100 for IF)	BD Biosciences	catalog number: 610051; RRID:AB_397463
Rabbit polyclonal antibody raised against Phospho-p130Cas (BCAR1, Tyr410)	Cell signaling	catalog number: 4011; RRID:AB_2274823
Rabbit monoclonal antibody raised against human ILK (clone EPR1592; 1:100 for IF)	Abcam	catalog number: ab76468; RRID:AB_2126930

**Chemicals, Peptides, and Recombinant Proteins**		

SiR-actin	Cytoskeleton	catalog number: CY-SC001
Bovine plasma fibronectin	Merck	catalog number: 341631
Dimethylsulphoxide (DMSO)	Sigma-Aldrich	catalog number: D2650
SRC inhibitor (PP2)	Selleckchem	catalog number: S7008
FAK (PTK2) inhibitor (PF-573228)	Selleckchem	catalog number: S2013
The myosin II inhibitor blebbistatin	STEMCELL Technologies	catalog number: 72402
Lipofectamine 3000 and the P3000TM Enhancer Reagent	Thermo Fisher Scientific	catalog number: L3000015
Human EGF	Sigma-Aldrich	catalog number: E9644
Hydrocortisone	Sigma-Aldrich	catalog number: H0888-1G
Cholera toxin	Sigma-Aldrich	catalog number: C8052-1MG
Insulin	Sigma-Aldrich	catalog number: I9278-5ML
Penicillin/streptomycin	Sigma-Aldrich	catalog number: P0781-100ML
Silane A-174	Sigma-Aldrich	catalog number: 440159-100ML
Acrylamide solution	Sigma-Aldrich	catalog number: A4058
N, N′-Methylenebisacrylamide solution	Sigma-Aldrich	catalog number: M1533
TEMED	Sigma-Aldrich	catalog number: T9281
Sulfo-SANPAH	Thermo Scientific	catalog number: 22589
N-(3-Dimethylaminopropyl)-N’ ethylcarbodiimide hydrochloride (EDC)	Sigma-Aldrich	catalog number: 03450

**Experimental Models: Cell Lines**		

U2OS osteosarcoma cells	Leibniz Institute DSMZ-German Collection of Microorganisms and Cell Cultures, Braunschweig DE	**catalog number: ACC 785**
MDA-MB-231 triple-negative human breast adenocarcinoma	ATCC	catalog number: HTB-26
MCF10 DCIS.COM	**J.F. Marshall (Barts Cancer Institute, Queen Mary University of London, London, England, UK) [**57**]**	N/A
RAT2 cells (Rat embryonic fibroblasts)	ATCC	catalog number: CRL-1764

**Oligonucleotides**		

The siRNA used as control (siCTRL) was Allstars negative control siRNA.	QIAGEN	catalog number: 1027281
siBCAR1#5 (Hs_BCAR1_5)	QIAGEN	catalog number: SI02757734
siBCAR1#6 (Hs_BCAR1_6)	QIAGEN	catalog number: SI02757741

**Recombinant DNA**		

eGFP-TNS1	Previous study [58]	N/A
eGFP-TNS2	Previous study [58]	N/A
eGFP-TNS3	Previous study [59]	N/A
eGFP-TNS4	Previous study [58]	N/A
GFP-Sharpin	Previous study [60]	N/A
GFP-MDGI	Previous study [61]	N/A
GFP-FAK-FAT	David D. Schlaepfer (UC San Diego Health, US)	N/A
GFP-FL-FAK	David D. Schlaepfer (UC San Diego Health, US)	N/A
EFHD2-GFP	Dirk Mielenz (University of Erlangen-Nuremberg, DE) [62]	N/A
CAAX-GFP	Gregory Giannone (Bordeaux University, FR)	N/A
EGFP-Talin-1	Ben Goult (University of Kent, UK)	N/A
mCherry-Talin-2	Ben Goult (University of Kent, UK)	N/A
pDsRedC1-Kindlin-1	Ben Goult (University of Kent, UK)	N/A
FMNL2-GFP	Robert Grosse (University of Marburg, DE) [63]	N/A
FMNL3-GFP	Henry Higgs (Geisel School of Medicine at Dartmouth, US) [18]	N/A
PPFIA1-GFP	Guido Serini (University of Torino, IT)	N/A
pEGFP-C1-Lamellipodin	Matthias Krause (King’s College London, UK) [19]	N/A
pEGFP-C2-Myo15a	Jonathan Bird (NIH, Bethesda US) [64]	N/A
GFP-ICAP-1	Daniel Bouvard (University of Grenoble, FR)	N/A
GFP-KANK1	Reinhard Fässler (Max Planck Institute of Biochemistry, Martinsried, DE) [65]	N/A
GFP-KANK2	Reinhard Fässler (Max Planck Institute of Biochemistry, Martinsried, DE) [65]	N/A
GFP-KANK3	Reinhard Fässler (Max Planck Institute of Biochemistry, Martinsried, DE) [65]	N/A
GFP-KANK4	Reinhard Fässler (Max Planck Institute of Biochemistry, Martinsried, DE) [65]	N/A
BTK-PH-EGFP	Matthias Wymann (University of Basel, Switzerland)	N/A
PLC(δ1)-PH-EGFP	Matthias Wymann (University of Basel, Switzerland)	N/A
EGFP-tagged tandem FYVE	Harald Stenmark (Oslo University Hospital) [66]	N/A
GFP-Cas-wt	Daniel Rösel (Charles University in Prague, Czech Republic) [37]	N/A
GFP-CasdeltaCCH	Daniel Rösel (Charles University in Prague, Czech Republic) [37]	N/A
GFP-CasdeltaSH3	Daniel Rösel (Charles University in Prague, Czech Republic) [37]	N/A
GFP-Cas-deltaCCH-deltaSH3	Daniel Rösel (Charles University in Prague, Czech Republic) [37]	N/A
Kindlin-2-GFP	Maddy Parsons (King’s College London, UK)	N/A
Ezrin-GFP	Maddy Parsons (King’s College London, UK)	N/A
Vinculin-GFP	Maddy Parsons (King’s College London, UK)	N/A
Moesin-GFP	Buzz Baum (University College London, UK)	N/A
Lifeact-mTurquoise2	Joachim Goedhart (University of Amsterdam, NL) [67]	N/A
Integrin alpha5-GFP	Rick Horwitz (Allen institute for cell science, US)	N/A
mEmerald-Alpha-Actinin-19	Addgene (Michael Davidson)	catalog number: 53989
mEmerald-Fascin-C-10	Addgene (Michael Davidson)	catalog number: 54094
pGFP Cas	Addgene (Kenneth Yamada)	catalog number: 50729
mEmerald-Cofilin-C-10	Addgene (Michael Davidson)	catalog number: 54047
mEmerald-Coronin1B-C-10	Addgene (Michael Davidson)	catalog number: 54049
pGFP CrkII	Addgene (Kenneth Yamada)	catalog number: 50730
mEmerald-Cortactin-C-12	Addgene (Michael Davidson)	catalog number: 54051
mEmerald-mDia1-C-14	Addgene (Michael Davidson)	catalog number: 54156
mEmerald-mDia2-C-14	Addgene (Michael Davidson)	catalog number: 54158
mEmerald-Migfilin-C-14	Addgene (Michael Davidson)	catalog number: 54181
pEGFP-IQGAP1	Addgene (David Sacks) [68]	catalog number: 30112
mEmerald-LASP1-C-10	Addgene (Michael Davidson)	catalog number: 54141
EGFP-EPLIN alpha	Addgene (Elizabeth Luna)	catalog number: 40947
EGFP-EPLIN beta	Addgene (Elizabeth Luna)	catalog number: 40948
mEmerald-PINCH-C-14	Addgene (Michael Davidson)	catalog number: 54229
mEmerald-MyosinIIA-C-18	Addgene (Michael Davidson) [69]	catalog number: 54190
mEmerald-MyosinIIB-C-18	Addgene (Michael Davidson)	catalog number: 54192
mEmerald-Palladin-C-7	Addgene (Michael Davidson)	catalog number: 54213
mEmerald-Parvin-C-14	Addgene (Michael Davidson)	catalog number: 54214
GFP-PTEN	Addgene (Alonzo Ross) [70]	catalog number: 13039
mEmerald-Paxillin-22	Addgene (Michael Davidson) [71]	catalog number: 54219
mEmerald-TES-C-14	Addgene (Michael Davidson)	catalog number: 54276
mEmerald-VASP-N-10	Addgene (Michael Davidson)	catalog number: 54297
mEmerald-Zyxin-6	Addgene (Michael Davidson)	catalog number: 54319
E-cadherin-GFP	Addgene (Jennifer Stow) [72]	catalog number: 28009
pEGFP C1-Eps8 WT	Addgene (Giorgio Scita) [73]	catalog number: 74950
GFP-P4M-SidM	Addgene (Tamas Balla) [74]	catalog number: 51469
pcDNA3.1-6His-MyoX	Addgene (Emanuel Strehler) [75]	catalog number: 47607
ARP3-GFP	Addgene (Matthew Welch) [76]	catalog number: 8462
mScarlet-MYO10	This study (Data S1)	N/A
pEGFP-CasCCHD	This study (Data S1)	N/A
mEmerald-MYO7A	This study	N/A
mEmerald-BAIAP2	This study	N/A
mEmerald-PDLIM5	This study	N/A
mEmerald-FERMT2	This study	N/A
mEmerald-FHL2	This study	N/A
mEmerald-FHL3	This study	N/A
mEmerald-PDLIM7	This study	N/A
mEmerald-PLS3	This study	N/A
mEmerald-TRIP6	This study	N/A
mEmerald-ALYREF	This study	N/A
mEmerald-ANXA1	This study	N/A
mEmerald-BRIX1	This study	N/A
mEmerald-DIMT1	This study	N/A
mEmerald-FAU	This study	N/A
mEmerald-HP1BP3	This study	N/A
mEmerald-PCOLCE	This study	N/A
mEmerald-POLDIP3	This study	N/A
mEmerald-SERPINE1	This study	N/A
mEmerald-SORBS1	This study	N/A
mEmerald-TGM2	This study	N/A
mEmerald-P4HB	This study	N/A
mEmerald-PDLIM1	This study	N/A
mEmerald-SYNCRIP	This study	N/A
mEmerald-NISCH	This study	N/A
mEmerald-PRSS23	This study	N/A

**Software and Algorithms**		

Filopodia mapping	This study (Data S2)	N/A
RStudio (1.0.153)	Foundation for Open Access Statistics.	https://www.rstudio.com/
DAVID platform	[77]	https://david.ncifcrf.gov/
BoxPlotR	[78]	http://shiny.chemgrid.org/boxplotr/
PlotsOfData	[79]	https://huygens.science.uva.nl/PlotsOfData/
Fiji	[80]	https://fiji.sc/
Fiji plugin TrackMate	[81]	https://imagej.net/TrackMate
MATLAB (R2018b)	MathWorks	https://se.mathworks.com/products/matlab.html
TFM analysis (MATLAB software)	Timo Betz (University of Münster) [82]	N/A

**Other**		

Glass-bottom dishes, High Tolerance 1.5 coverslip	MatTek Corporation	catalog number: **P35G-0.170-14-C**
Glass-bottom dishes, 1.0 coverslip	MatTek Corporation	catalog number: P35G-1.0-14-C
Polyacrylamide gels of defined stiffness; 0.5 kPa	Matrigen	catalog number: SV3510-EC-0.5
Polyacrylamide gels of defined stiffness; 50 kPa	Matrigen	catalog number: SV3510-EC-50
VECTASHIELD	Vector laboratories	catalog number: H-1000
FluoSpheres (505/515)	Life Technologies	catalog number: F881
Horse serum	GIBCO BRL	catalog number: 16050-122

### Contact for Reagent and Resource Sharing

Further information and requests for resources and reagents should be directed to and will be fulfilled by the Lead Contact, Johanna Ivaska (johanna.ivaska@utu.fi).

### Experimental Model and Subject Details

U2OS osteosarcoma cells, MDA-MB-231 (triple-negative human breast adenocarcinoma) cells and RAT2 cells (Rat embryonic fibroblasts) were grown in DMEM supplemented with 10% FCS. MCF10 DCIS.COM (DCIS.COM) cells were cultured in a 1:1 mix of DMEM (Sigma-Aldrich) and F12 (Sigma-Aldrich) supplemented with 5% horse serum (16050-122; GIBCO BRL), 20 ng/ml human EGF (E9644; Sigma-Aldrich), 0.5 mg/ml hydrocortisone (H0888-1G; Sigma-Aldrich), 100 ng/ml cholera toxin (C8052-1MG; Sigma-Aldrich), 10 μg/ml insulin (I9278-5ML; Sigma-Aldrich), and 1% (vol/vol) penicillin/streptomycin (P0781-100ML; Sigma-Aldrich). U2OS cells were purchased from DSMZ (Leibniz Institute DSMZ-German Collection of Microorganisms and Cell Cultures, Braunschweig DE, ACC 785). MDA-MB-231 and RAT2 cells were provided by ATCC. DCIS.COM were provided by J.F. Marshall (Barts Cancer Institute, Queen Mary University of London, London, England, UK). All cells were tested for mycoplasma contamination. Cell lines have not been authenticated.

### Method Details

#### Plasmids and transfection

Plasmids of interest were transfected using Lipofectamine 3000 and the P3000TM Enhancer Reagent (Thermo Fisher Scientific) according to the manufacturer’s instructions. The expression of proteins of interest was suppressed using 100 nM siRNA and lipofectamine 3000 (ThermoFisher Scientific) according to manufacturer’s instructions. The siRNA used as control (siCTRL) was Allstars negative control siRNA (QIAGEN, Cat. No. 1027281). The siRNAs targeting BCAR1 were purchased from QIAGEN (siBCAR1#5, Hs_BCAR1_5 FlexiTube siRNA, Cat. No. SI02757734; siBCAR1#6, Hs_BCAR1_6 FlexiTube siRNA, Cat. No. SI02757741).

#### Plasmids

The mScarlet-MYO10 construct was generated by inserting a gene block containing the mScarlet sequence (IDT, see Data S2 for sequence) [83] into pcDNA3.1-6His-MyoX using the KpnI restriction site. The BCAR1 CCHD construct was generated by inserting a gene block containing the BCAR1 CCHD sequence (IDT, see see Data S2 for sequence) into pEGFP-C1 using the XhoI and BamHI restriction sites. Several of the GFP tagged constructs, used here, were generated by the Genome Biology Unit core facility cloning service (Research Programs Unit, HiLIFE Helsinki Institute of Life Science, Faculty of Medicine, University of Helsinki, Biocenter Finland) by transferring entry clones from the ORFeome collaboration library into mEmerald destination vectors using the standard LR reaction protocol. Entry clone (I.M.A.G.E. Consortium CloneID [68],) transferred into pcDNA6.2/N-emGFP-DEST include MYO7A (100069043), BAIAP2 (100006086), PDLIM5 (100003316), FERMT2 (100067023), FHL2 (100006502), FHL3 (100003225), PDLIM7 (100004765), PLS3 (100003800), TRIP6 (100005523), ALYREF (100066457), ANXA1 (100003803), BRIX1 (100004738), DIMT1 (100004112), FAU (100005039), HP1BP3 (100067555), PCOLCE (100004553), POLDIP3 (100000169), SERPINE1 (100003324) and SORBS1 (100064166). Entry clones transferred into pcDNA6.2/C-emGFP-DEST include TGM2 (100074142), P4HB (100073368), PDLIM1 (100069996), SYNCRIP (100074128), NISCH (100068156) and PRSS23 (100002084).

#### SDS–PAGE and quantitative western blotting

Protein extracts were separated under denaturing conditions by SDS–PAGE and transferred to nitrocellulose membranes. Membranes were blocked for 1 h at room temperature with blocking buffer (LI-COR Biosciences) and then incubated overnight at 4°C with the appropriate primary antibody diluted in blocking buffer. Membranes were washed with PBS and then incubated with the appropriate fluorophore-conjugated secondary antibody diluted 1:5,000 in blocking buffer for 30 min. Membranes were washed in the dark and then scanned using an Odyssey infrared imaging system (LI-COR Biosciences). Band intensity was determined by digital densitometric analysis using the Odyssey software.

### Sample preparation for light microscopy

If not indicated otherwise, cells were plated on high tolerance glass-bottom dishes (MatTek Corporation, coverslip #1.7) pre-coated first with Poly-L lysine (10 μg/ml, 1 h at 37°C) and then with bovine plasma fibronectin (10 μg/ml, 2 h at 37°C).

To generate the filopodia map, U2OS cells transiently expressing a GFP-tagged protein of interest (POI) and MYO10-mScarlet were plated for 2 h on fibronectin-coated glass-bottom dishes. Samples were fixed and permeabilised simultaneously using a solution of 4% (wt/vol) PFA and 0.25% (vol/vol) Triton X-100 for 10 min. Cells were then washed with PBS, quenched using a solution of 1 M glycine for 30 min, and incubated with SiR-actin (100 nM in PBS) at 4°C until imaging (minimum length of staining, overnight at 4°C; maximum length, 1 week). Just before imaging, samples were washed three times in PBS and mounted in vectashield (Vectorlabs).

To stain endogenous proteins, U2OS cells transiently expressing MYO10-mScarlet were plated on fibronectin-coated glass-bottom dishes for 2h. Samples were fixed and permeabilised simultaneously using a solution of 4% (wt/vol) PFA and 0.25% (vol/vol) Triton X-100 for 10 min. Cells were then washed with PBS, quenched using a solution of 1 M glycine for 30 min, and incubated with the primary antibody for 1 h (1:100). After three washes, cells were incubated with a secondary antibody for 1 h (1:100). Samples were then washes three times and stored in PBS or in PBS containing an actin stain (as indicated) at 4°C until imaging. Just before imaging, samples were washed three times in PBS and mounted in vectashield (Vectorlabs).

For the filopodia formation assays, cells expressing human MYO10-GFP or MYO10-mScarlet were plated for 2 h in full medium either on glass-bottom dishes (MatTek Corporation) or on polyacrylamide gels of defined stiffness (Matrigen; 0.5 kPa, soft, SV3510-EC-0.5; 50 kPa, stiff, SV3510-EC-50) precoated with fibronectin (10 μg/ml). Cells were then fixed using PFA, washed with PBS, permeabilized and stained using phalloidin. Images were acquired using either an SDC microscope (100x objective) or an Airyscan confocal microscope (long-working-distance 63 × water objective). The number of filopodia per cell was manually scored using Fiji [80, 84]. Filopodia length were measured automatically using a custom-made Fiji plug-in as previously described [15].

#### Live cell imaging

All live-cell imaging experiments were performed in normal growth media, supplemented with 50 mM HEPES, at 37°C and in the presence of 5% CO_2_.

Dynamics of PI(3,4)P_2_, PXN, TLN1, ITGA5 and FMNL3 were recorded using an Airyscan microscope and a 40x objective.

To study the role of BCAR1 in filopodia stability, U2OS cells expressing MYO10-GFP were plated for at least 2 h on fibronectin before the start of live imaging (pictures taken every 5 s at 37°C, on an Airyscan microscope using a 40x objective). To study the role of extracellular stiffness in filopodia stability, U2OS cells expressing MYO10-GFP were plated for at least 2 h on polyacrylamide gels of defined stiffness (Matrigen; 0.5 kPa, soft, SV3510-EC-0.5; 50 kPa, stiff, SV3510-EC-50) precoated with fibronectin (10 μg/ml) before the start of live imaging (pictures taken every 5 s at 37°C, on an Airyscan microscope using a long-working-distance 63 × water objective). Filopodia lifetimes were then measured by identifying and tracking all MYO10 spots using the Fiji plugin TrackMate [81]. In Trackmate, the LoG detector (estimated bob diameter = 0.8 μm; threshold = 20; subpixel localization enabled) and the simple LAP tracker (linking max distance = 1 μm; gap-closing max distance = 1 μm; gap-closing max frame gap = 0) were used.

#### Traction force microscopy (TFM)

TFM analysis is performed by recording the position of fluorescent beads that are incorporated into a deformable gel in the presence and in the absence of cells. Bead displacement is then used to calculate the amount of force generated by cells onto the substrate.

Hydrogels used for TFM analyses were prepared as follows: glass-bottom dishes (MatTek, P35G-1.0-14-C) were treated with a solution of bind-silane (Sigma-Aldrich, Silane A-174) for 15 min at RT, washed once with 95% EtOH, twice with mQH_2_O before being left to dry. A pre-mixture composed of 94 μl of 40% acrylamide (Sigma, A4058), 50 μl of 2% N, N′-Methylenebisacrylamide solution (Sigma, M1533), and 356 μl of PBS was prepared (yielding a stiffness of ∼10kPa [59]). followed by addition of 3.4 μL of sonicated FluoSpheres (505/515) (Life Technologies, F881). The pre-mixture was then vortexed briefly, and 1/500 of TEMED (Sigma, T9281) and 1/100 APS 10% was added immediately before 11.7 μL of the mixture was pipetted onto the glass plates. A round 13 mm coverslip was carefully placed on top of the drop, ensuring that a thin layer of liquid remained between the two glass surfaces, and incubated for 30 min. The plate was then submerged in PBS and the glass coverslip was carefully removed. For functionalization, Sulfo-SANPAH (Thermo Scientific, 22589) 0.2 mg/ml and N-(3-Dimethylaminopropyl)-N’ ethylcarbodiimide hydrochloride (EDC) (Sigma, 03450) 2 mg/ml in 50 mM HEPES were added onto the gels and incubated for 30 min at RT with gentle agitation. Gels were placed into a UV-chamber for 10 min without cover for polymerization, washed three times with PBS and then coated with fibronectin at +4°C overnight.

Cells were plated onto TFM plates 2-4 h before the experiment. Cells were imaged live using a spinning disk confocal microscope (long-working-distance 63 × water objective). A fluorescence image of the beads (Excitation, 488 nm; Detection, 500–550 nm) and a phase-contrast image of the cells were recorded. To acquire a reference image (beads position in absence of cells), cells were removed by adding 20% SDS. Bead displacement was analyzed using a MATLAB (MathWorks) software package, kindly provided by Timo Betz (University of Münster), that employs a correlation algorithm previously described [82]. Traction stresses were determined using the same MATLAB software package following the Fourier transform traction force algorithm [85].

#### Microscopy setup

The structured illumination microscope (SIM) used was DeltaVision OMX v4 (GE Healthcare Life Sciences) fitted with a 60 × Plan-Apochromat objective lens, 1.42 NA (immersion oil RI of 1.516) used in SIM illumination mode (five phases × three rotations). Emitted light was collected on a front illuminated pco.edge sCMOS (pixel size 6.5 μm, readout speed 95 MHz; PCO AG) controlled by SoftWorx.

The spinning disk microscope used was a Marianas spinning disk imaging system with a Yokogawa CSU-1 scanning unit on an inverted Zeiss Axio Observer Z1 microscope controlled by SlideBook 6 (Intelligent Imaging Innovations). Objectives used were a long-working-distance 63 × water (NA 1.15 water, LDC-Apochromat, M27) objective or a 100 × (NA 1.4 oil, Plan-Apochromat, M27) objective. Images were acquired using either an Orca Flash4 sCMOS camera (chip size 2,048 × 2,048; Binning 2x2 enabled; Hamamatsu Photonics) or an Evolve 512 EMCCD camera (chip size 512 × 512; Photometrics).

The confocal microscope used was a laser scanning confocal microscope LSM880 (Zeiss) equipped with an Airyscan detector (Carl Zeiss). Objectives used were a long-working-distance 63 × water (NA 1.15 water, LDC-Apochromat, M27) or a 40x oil (1.4). The microscope was controlled using Zen Black (2.3) and the Airyscan was used in standard super-resolution mode.

#### Mapping of proteins within filopodia

To map the localization of each POI within filopodia, images were first processed in Fiji [80] and data analyzed using R. Briefly, in Fiji, the brightness and contrast of each image was automatically adjusted using, as an upper maximum, the brightest cellular structure labeled in the field of view. In Fiji, line intensity profiles (1 pixel width) were manually drawn from filopodium tip to base (defined by the intersection of the filopodium and the lamellipodium). To avoid any bias in the analysis, the intensity profile lines were drawn from a merged image of actin, MYO10, and POI. All visible filopodia in each images were analyzed and exported for further analysis (export was performed using the “Multi Plot” function). Data S1 contains the number of filopodia and cells analyzed for each POI. The Fiji script used to process the data is available as supplementary information (Data S2). For each POI, line intensity profiles were then compiled and analyzed in R. To homogenize filopodia length, each line intensity profile was binned into 40 bins (using the median value of pixels in each bin and the R function “tapply”). Using the line intensity profiles, the percentage of filopodia positive for each POI was quantified. A positive identification was defined as requiring at least three bins (out of 40), each with a minimum value of 10000 (bin values between 0-65535). The map of each POI was created by averaging hundreds of binned intensity profiles. The R script used to bin and average line intensity profiles is available as supplementary information (Data S2). The averaged binned intensity profiles of each POI is available in Data S2. The filopodia maps were then displayed as heatmaps in R (Data S2).

The length of each filopodia analyzed were directly extracted from the line intensity profiles and are plotted in Figure S1.

The preferential recruitment of core filopodia proteins to filopodia tips or shafts was assessed by calculating an enrichment ratio where the averaged intensity of a POI at filopodium tip (bin 1-6) was divided by the averaged intensity of a POI at filopodia shaft (bin 7-40). This enrichment ratio was calculated for each filopodium analyzed and the results are displayed as Tukey boxplots in Figure 4.

The functional annotation analysis (protein domain enrichments and search for known interactors) of the core filopodia proteins was performed using the DAVID platform [77].

### Quantification and Statistical Analysis

The images used to generate the filopodia map were acquired from two independent experiments. All other experiments were replicated at least three times. No strategy was employed for randomization and/or stratification. No blinding or sample-size estimations were performed at any stage of the study. No data were excluded from the analyses.

The Tukey boxplots represent the median and the 25^th^ and 75^th^ percentiles (interquartile range); points are displayed as outliers (represented by dots) if 1.5 times above or below the interquartile range (represented by whiskers). Boxplots were generated using the online tool BoxPlotR (http://shiny.chemgrid.org/boxplotr/) [78]. Violin plots and dot plots were generated using the online tool PlotsOfData (https://huygens.science.uva.nl/PlotsOfData/) [79].

Statistical analyses were performed when appropriate, and p values are indicated in the figure legends. Unless otherwise indicated, the Student’s t test was used (unpaired, two tailed, and unequal variance, performed within LibreOffice Calc).

### Data and Software Availability

Representative images highlighting the subcellular localization of each protein of interest (POI) to generate the filopodia map are available in Data S2. Data S2 also contains the scripts used for generating the map. Script 1 is the ImageJ macro used to measure and export the line intensity profiles from filopodia. Script 2 is the R code used to extract, compile, and average the line intensity profiles previously measured in ImageJ. Script 3 is the R code used to generate the filopodia map (using the “input table.csv” file) displayed in Figure 2. The “input table.csv” file contains the numerical values used to generate the filopodia map displayed in Figure 2.

## Supplemental Information

Document S1. Figures S1–S6

Data S1. Constructs Imaged to Generate the Filopodia Map, Related to Figures 1, 2, 3, 4, and 6 Data S1 highlights the various proteins / constructs imaged in this study as well as the number of filopodia analyzed to generate Figures 1, 2, 3, 4, and 7.

Data S2. Resources Used to Generate the Filopodia Map, Related to Figures 1, 2, 3, 4, and 6 Data S2 contains a PDF file where representative images highlight the subcellular localisation of each protein of interest (POI) to generate the filopodia map (Figure 2). Data S2 also contains the scripts used for generating the map. Script 1 is the ImageJ macro used to measure and export the line intensity profiles from filopodia. Script 2 is the R code used to extract, compile and average the line intensity profiles previously measured in ImageJ. Script 3 is the R code used to generate the filopodia map (using the “input table.csv” file) displayed in Figure 2. The “input table.csv” file contains the numerical values used to generate the filopodia map displayed in Figure 2.

Document S2. Article plus Supplemental Information
