# Supplementary material for: Filopodome Mapping Identifies p130Cas as a Mechanosensitive Regulator of Filopodia Stability
Source: Curr Biol. 2019 Jan 21;29(2):202–216.e7. doi: 10.1016/j.cub.2018.11.053 (PMC6345628; doi:10.1016/j.cub.2018.11.053)
Supplement: Document S1. Figures S1–S6 [file mmc1.pdf]

Current Biology, Volume *29*

Supplemental Information

Filopodome Mapping Identifies p130Cas as a Mechanosensitive Regulator of Filopodia Stability

Guillaume Jacquemet, Aki Stubb, Rafael Saup, Mitro Miihkinen, Elena Kremneva, Hellyeh Hamidi, and Johanna Ivaska

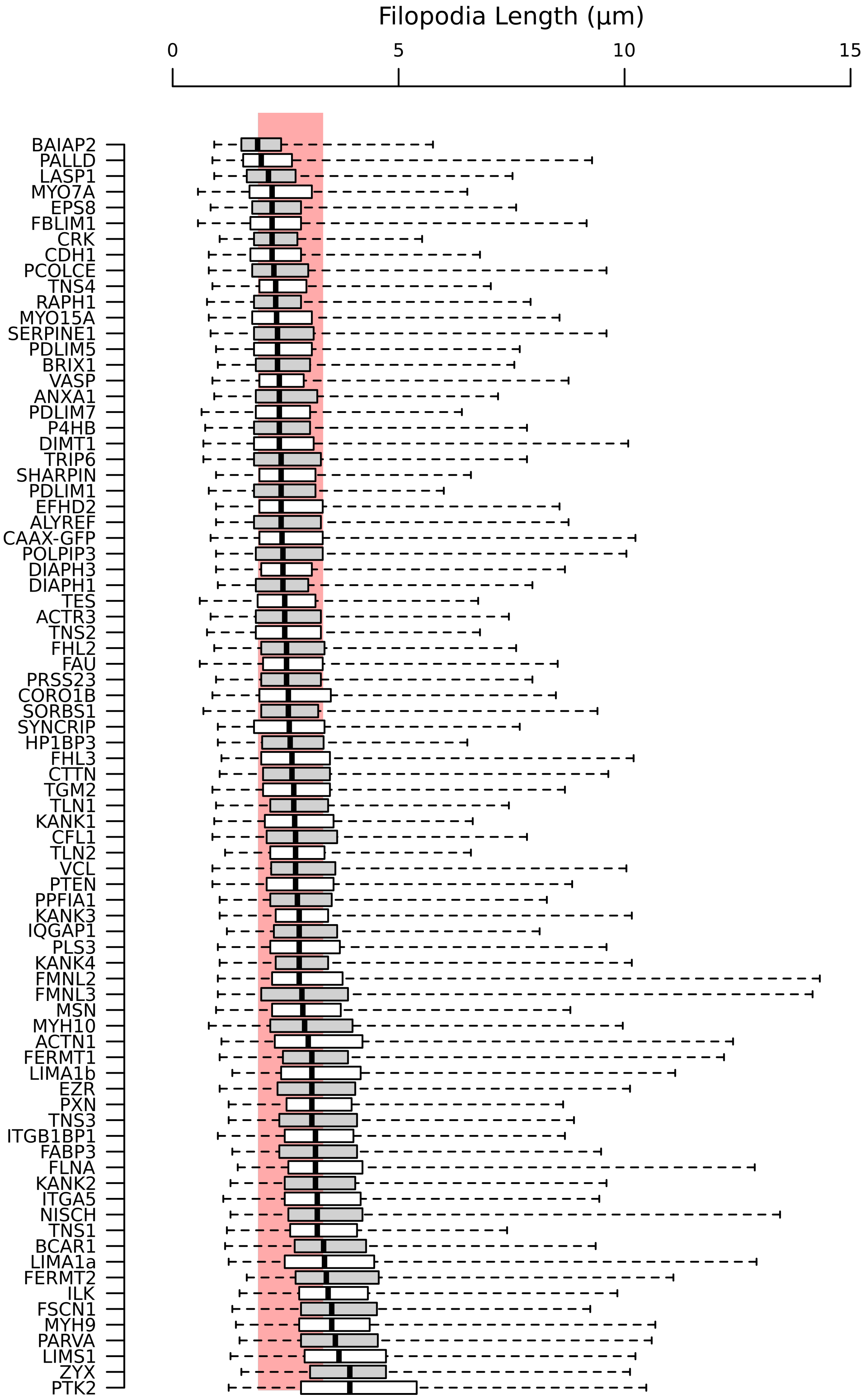

Figure S1: Length of the filopodia analysed to generate the filopodia ma p. Related to Figure 2. To map the localisation of 80 key adhesion proteins within filopodia, U2OS cells expressing a GFP/RFP-tagged POI and GFP/RFP-MYO10 were plated on fibronectin for 2 h, stained for F-actin and imaged using SIM. POI distribution within filopodia (from tip to base) was assessed with line intensity profiles (n numbers can be found in Data S1). Here, the length of the filopodia analysed to generate the filopodia map in Figure 2 are displayed as Tukey box plots. The POI are ordered as a function of median filopodia length. The filopodia length measured in cells expressing CAAX-GFP is highlighted in red.

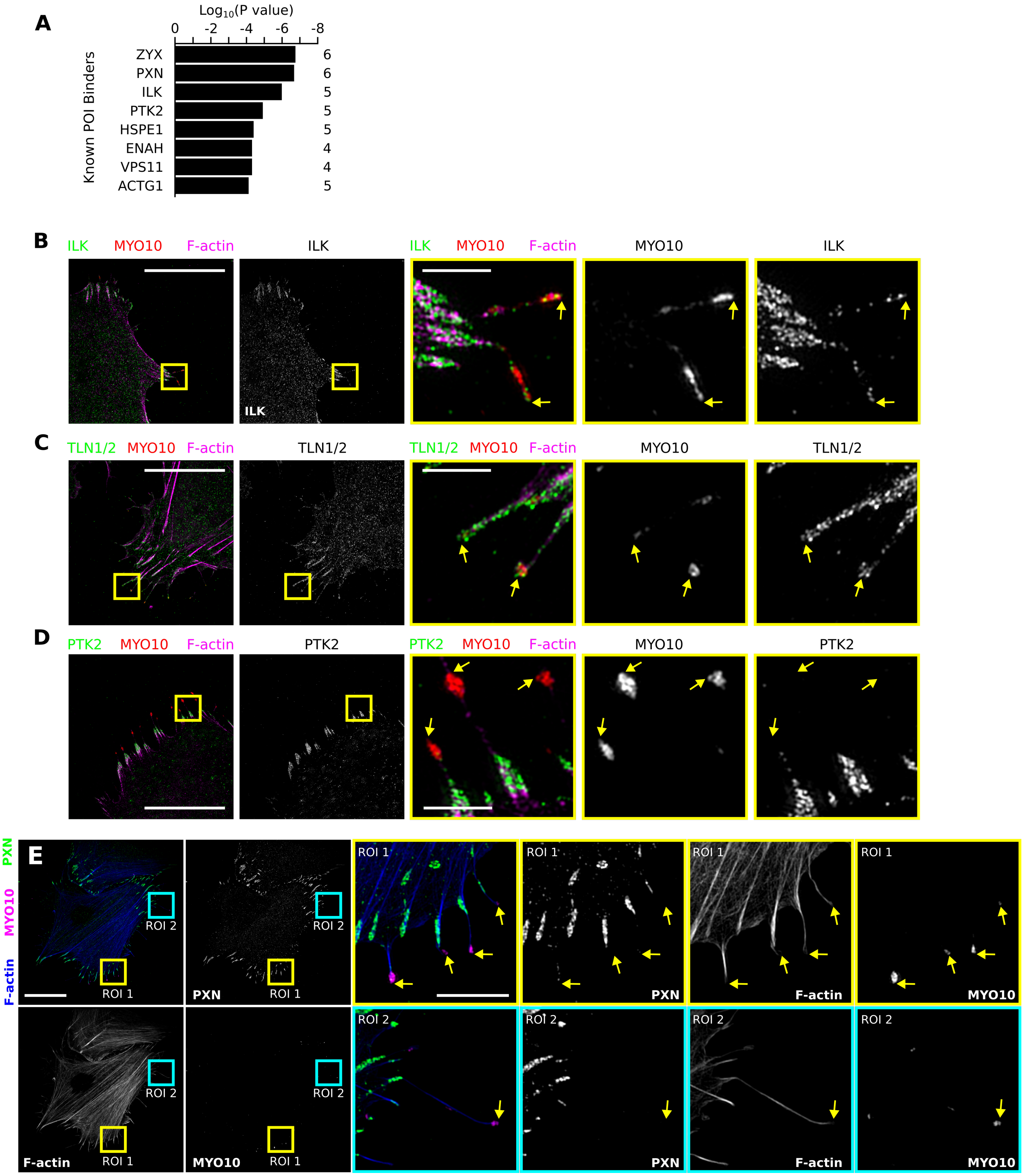

Figure S2: Endogenous staining of adhesion proteins in MYO10-induced filopodia. Related to Figure 5. A: Functional annotation analysis searching for known interactors of the core filopodia proteins identified here. The interactors that were found to be significantly enriched (p-value; based on interaction with multiple core filopodia proteins) are shown (official human gene name) and the number of filopodia core protein interactions are indicated on the right-hand side. The analysis was performed using the BIOGRID database integrated within the DAVID platform. B-D: U2OS cells expressing MYO10-mScarlet were plated on fibronectin for 2h, fixed and stained for F-actin together with endogenous ILK (B), endogenous TLN1 (C) or endogenous PTK2 (D) before being imaged using SIM. Representative MIP are displayed; yellow and blue squares highlight ROI, which are magnified; yellow arrows highlight filopodia tips; scale bars: (main) 20 µm; (inset) 2 µm. E: U2OS cells expressing MYO10-mScarlet were plated on fibronectin for 2 h, fixed and stained for endogenous PXN and F-actin before being imaged using SIM. A representative MIP is displayed; blue and yellow squares highlight ROI, which are magnified; scale bars: (main) 20 µm; (inset) 5 µm.

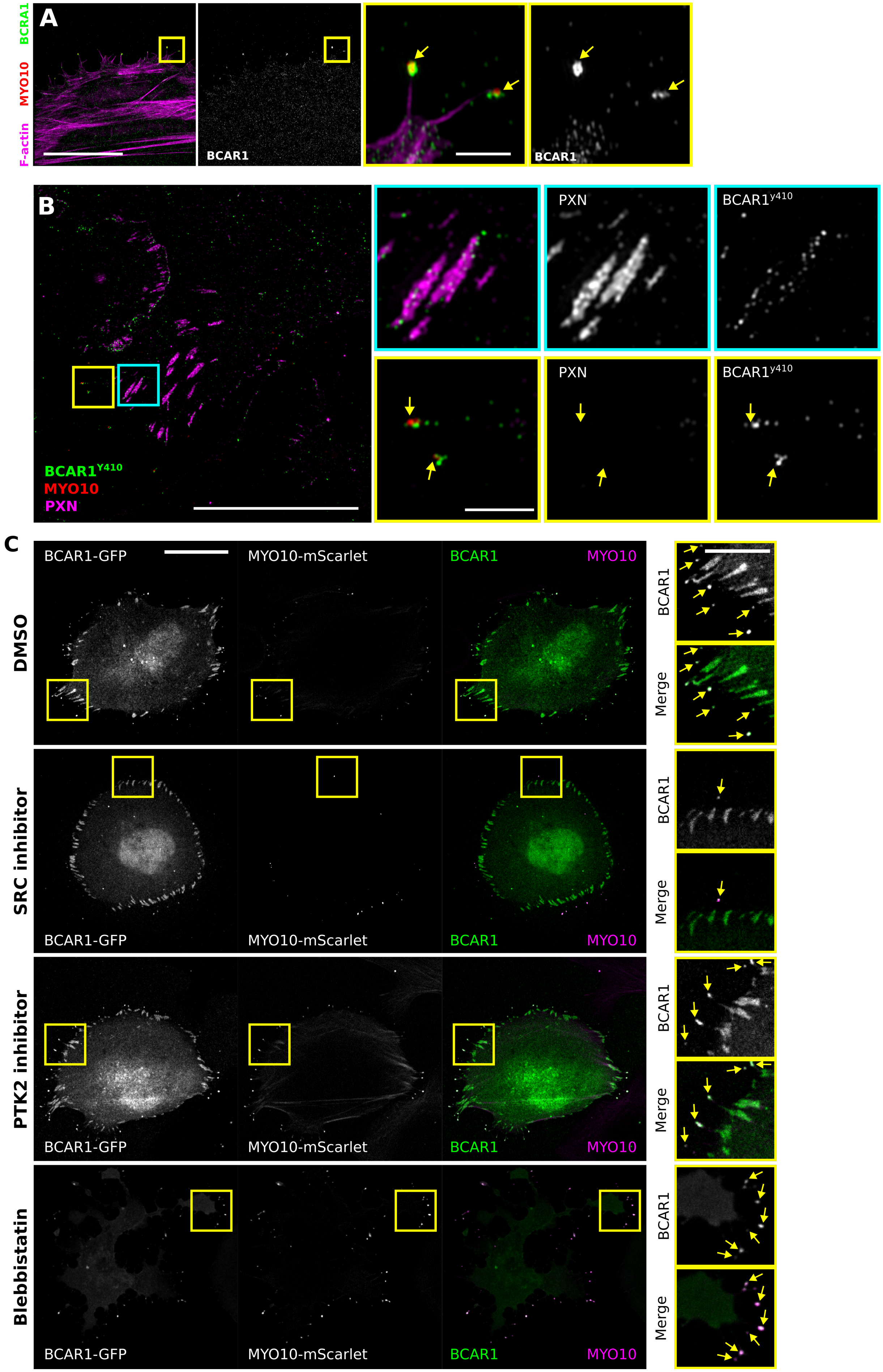

Figure S3: BCAR1 is part of the filopodia tip complex. Related to Figure 5. A-B: U2OS cells expressing MYO10-mScarlet were plated on fibronectin for 2 h, fixed and stained for F-actin and endogenous BCAR1 (A) or endogenous PXN and phospho-BCAR1 (Y410, B) before being imaged using SIM. Representative MIP are displayed; blue and yellow squares highlight ROI, which are magnified; scale bars: (main) 20 µm; (inset) 2 µm. C: U2OS cells expressing MYO10-mScarlet and BCAR1-GFP were plated on fibronectin for 1 h and then treated for 1 h with a SRC inhibitor (PP2, 10 µM), a PTK2 (FAK) inhibitor (PF-573228, 1 µM) or a myosin II inhibitor (blebbistatin, 10 µM). Cells were then fixed and imaged on a spinning disk confocal microscope (100x objective, EMCCD camera). Yellow squares highlight ROI, which are magnified; yellow arrows highlight filopodia tips; scale bars: (main) 25 µm; (inset) 10 µm.

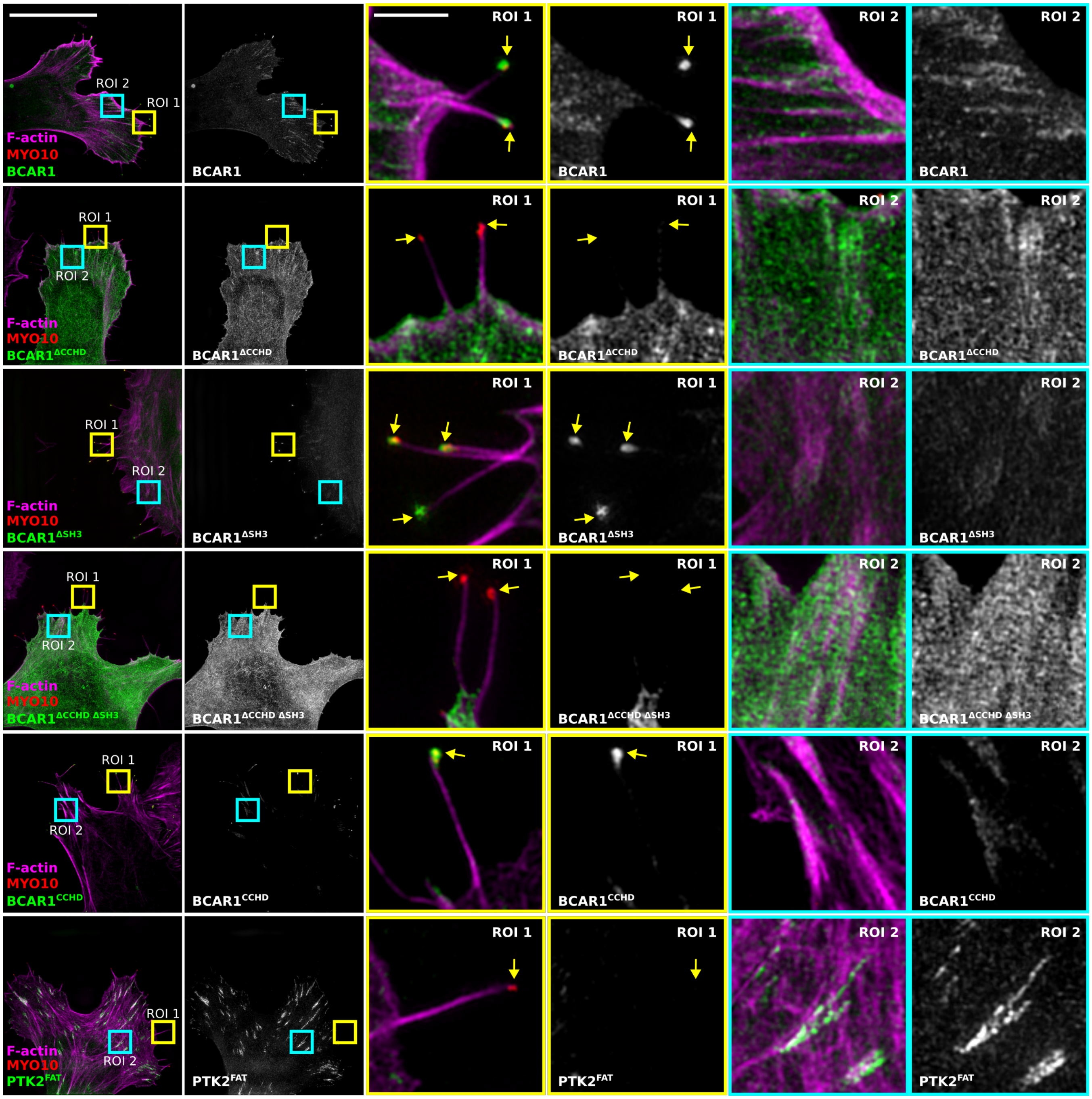

Figure S4: BCAR1 is recruited to filopodia tips via its CCHD domain. Related to Figure 5. U2OS cells expressing MYO10-mScarlet together with GFP tagged-BCAR1 or BCAR1 deletion constructs, or GFP-PTK2-FAT (PTK2FAT) were plated on fibronectin for 2 h, fixed, stained for F-actin and imaged using SIM. BCAR1$^{\Delta SH3}$: BCAR1 SH3 domain deletion; BCAR1$^{\Delta CCHD}$: BCAR1 CCHD domain deletion; BCAR1$^{\Delta CCHD \Delta SH3}$: BCAR1 SH3 and CCHD domain deletion; BCAR1^{CCHD}: BCAR1 CCHD domain alone. Representative MIP are displayed; yellow and blue squares highlight ROI, which are magnified; yellow arrows highlight filopodia tips; scale bars: (main) 20 µm; (inset) 2 µm.

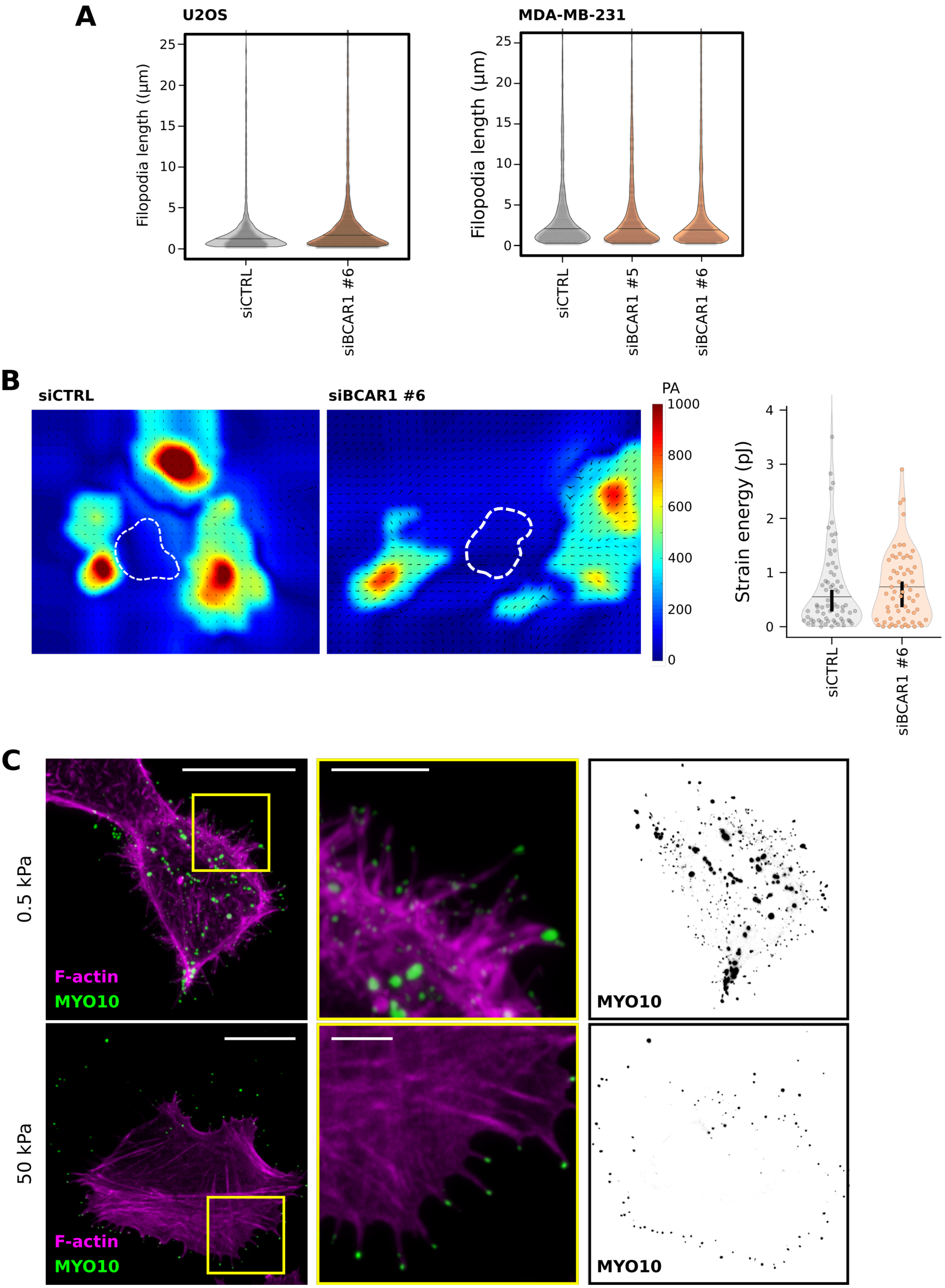

Figure S5: BCAR1, filopodia and substrate stiffness. Related to figure 6. A: BCAR1-silenced U2OS (oligo 6) and MDA-MB-231 (oligo 5 and 6) cells transiently expressing MYO10-GFP, were plated on fibronectin for 2 h, fixed and the length of MYO10-positive filopodia were measured (n > 65 cells, three biological repeats). Results are displayed as violin plots. B: BCAR-silenced U2OS cells (oligo 6) were plated on fibronectin-coated (10 µg/ml) polyacrylamide gels with a Young's modulus of 10 kPa and subjected to traction force microscopy analysis. Representative traction force heatmaps display the magnitude of traction stress in Pa units (pN/µm2) in a single cell for each condition. Black arrows indicate the direction of traction stress; white dotted lines denote nuclei contours. The forces (strain energy, pJ) exerted by control and BCAR1-silenced cells are displayed as violin plots (n > 62 cells, three biological repeats). C: U2OS cells expressing MYO10-GFP (from Figure 6G and 6H) were plated on fibronectin-coated polyacrylamide gels of defined stiffness (0.5 kPa, soft; 50 kPa, stiff) for 2 h. Cells were stained for F-actin and imaged using an Airyscan confocal microscope. Representative MIP are displayed. MYO10 spots observed on the soft substrate are located underneath the cell body. Scale bars: (main) 20 µm; (inset) 5 µm.

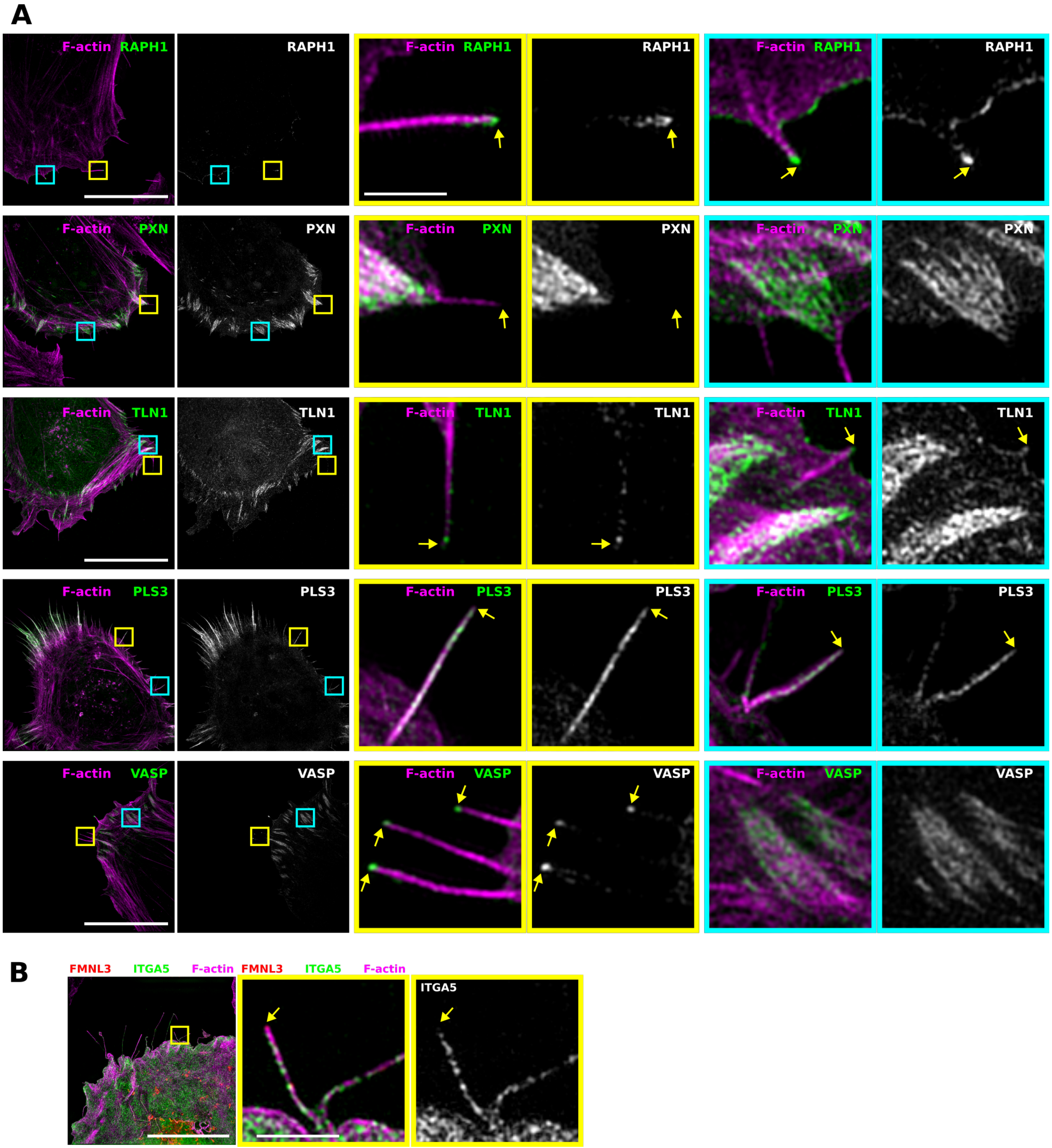

Figure S6: Adhesion proteins and endogenous filopodia. Related to figure 7. A: RAT2 cells expressing RAPH1-GFP, PXN-GFP, TLN1-GFP, PLS3-GFP or VASP-GFP were plated on fibronectin for 2 h, fixed and stained for F-actin before being imaged using SIM. Representative MIP are displayed; blue and yellow squares highlight ROI, which are magnified; scale bars: (main) 20 µm; (inset) 2 µm. B: U2OS cells expressing FMNL3-GFP and ITGA5-mCherry were plated on fibronectin for 2 h, fixed and stained for F-actin before being imaged using SIM. Representative MIP are displayed; the yellow square highlights an ROI, which is magnified; scale bars: (main) 20 µm; (inset) 2 µm.
