# Supplementary material for: Filopodome Mapping Identifies p130Cas as a Mechanosensitive Regulator of Filopodia Stability
Source: Curr Biol. 2019 Jan 21;29(2):202–216.e7. doi: 10.1016/j.cub.2018.11.053 (PMC6345628; doi:10.1016/j.cub.2018.11.053)
Supplement: Data S2. Resources Used to Generate the Filopodia Map, Related to Figures 1, 2, 3, 4, and 6 — Data S2 contains a PDF file where representative images highlight the subcellular localisation of each protein of interest (POI) to generate the filopodia map (Figure 2). Data S2 also contains the scripts used for generating the map. Script 1 is the ImageJ macro used to measure and export the line intensity profiles from filopodia. Script 2 is the R code used to extract, compile and average the line intensity profiles previously measured in ImageJ. Script 3 is the R code used to generate the filopodia map (using the “input table.csv” file) displayed in Figure 2. The “input table.csv” file contains the numerical values used to generate the filopodia map displayed in Figure 2. [file mmc9.zip › DataS1/DataS1.pdf]

Data S1

Data S1 (related to Figure 1 and 2) contains a PDF file where representative images highlight the subcellular localisation of each protein of interest (POI) to generate the filopodia map (Figure 2). Data S1 also contains the scripts used for generating the map. Script 1 is the ImageJ macro used to measure and export the line intensity profiles from filopodia. Script 2 is the R code used to extract, compile and average the line intensity profiles previously measured in ImageJ. Script 3 is the R code used to generate the filopodia map (using the "input table.csv" file) displayed in Figure 2. The "input table.csv" file contains the numerical values used to generate the filopodia map displayed in Figure 2.

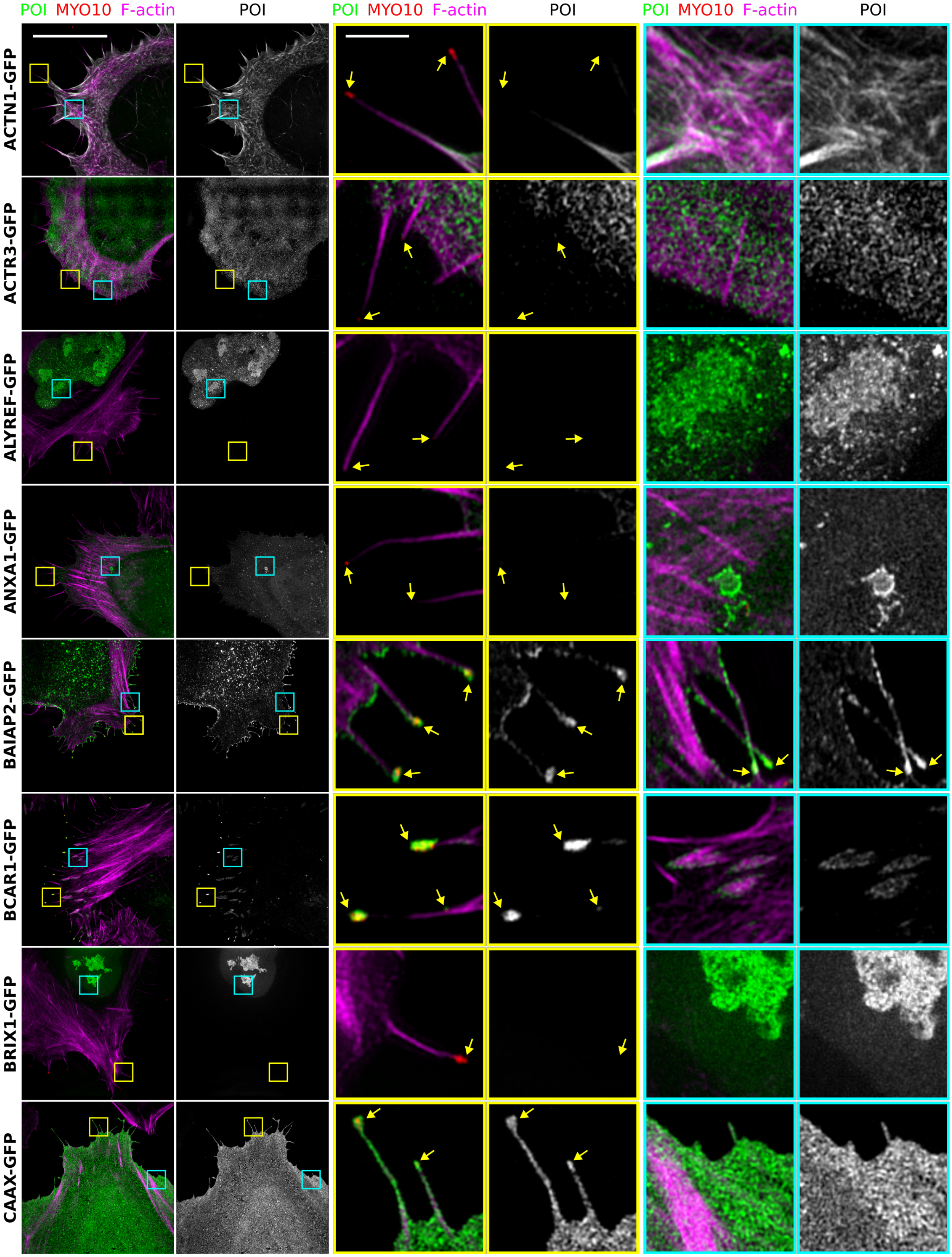

Representative images of each of the proteins imaged to generate the filopodia map. U2OS cells expressing GFP/RFP-tagged proteins of interest (POI, labelled with official human gene names) and GFP/RFP-MYO10 were plated on fibronectin for 2h, fixed and stained for F-actin before being imaged using SIM. A representative MIP is displayed for each condition. The blue and yellow squares highlight ROI, which are magnified; yellow arrows highlight filopodia tips; scale bars: (main) 20 µm; (inset) 2 µm.

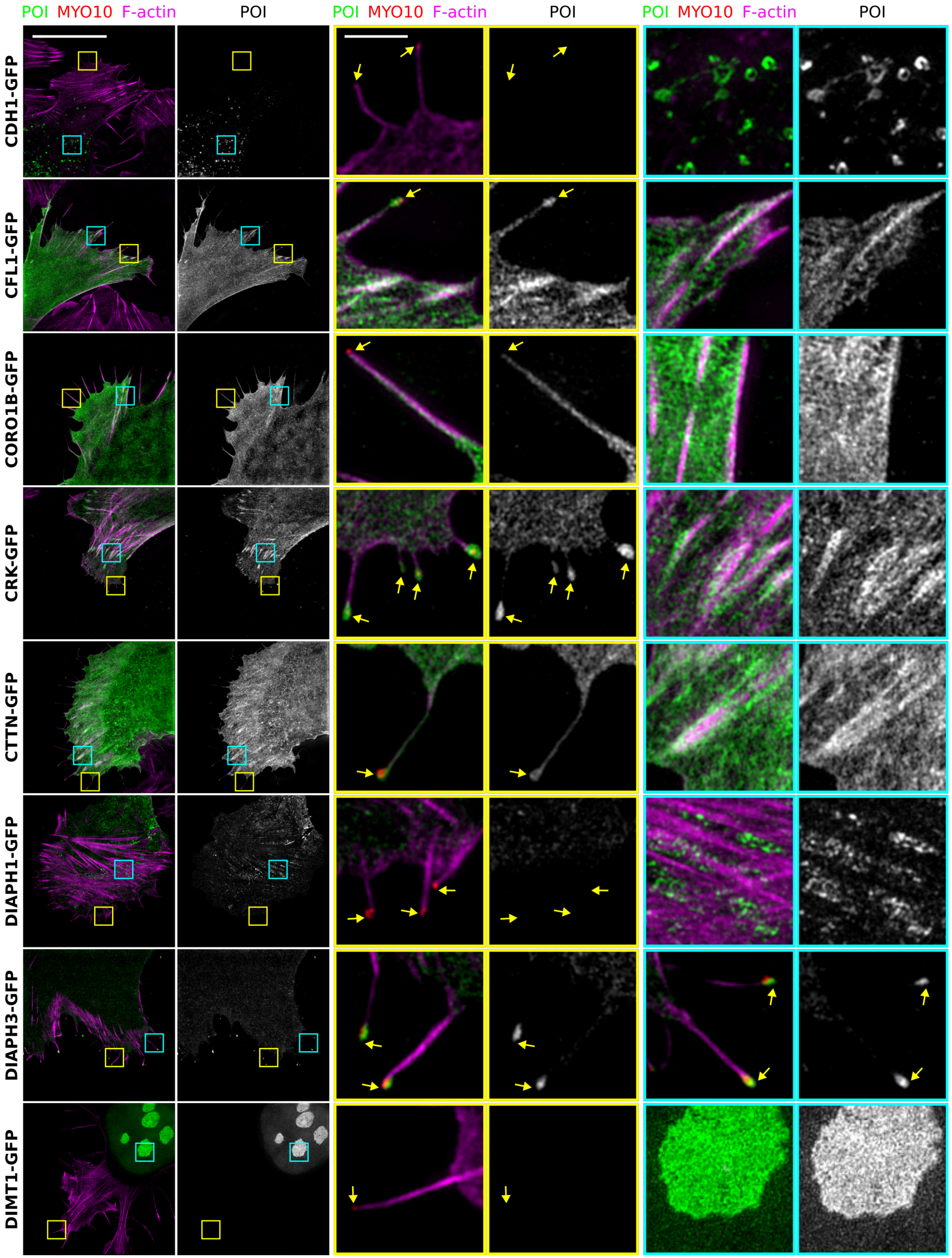

Representative images of each of the proteins imaged to generate the filopodia map. U2OS cells expressing GFP/RFP-tagged proteins of interest (POI, labelled with official human gene names) and GFP/RFP-MYO10 were plated on fibronectin for 2h, fixed and stained for F-actin before being imaged using SIM. A representative MIP is displayed for each condition. The blue and yellow squares highlight ROI, which are magnified; yellow arrows highlight filopodia tips; scale bars: (main) 20 µm; (inset) 2 µm.

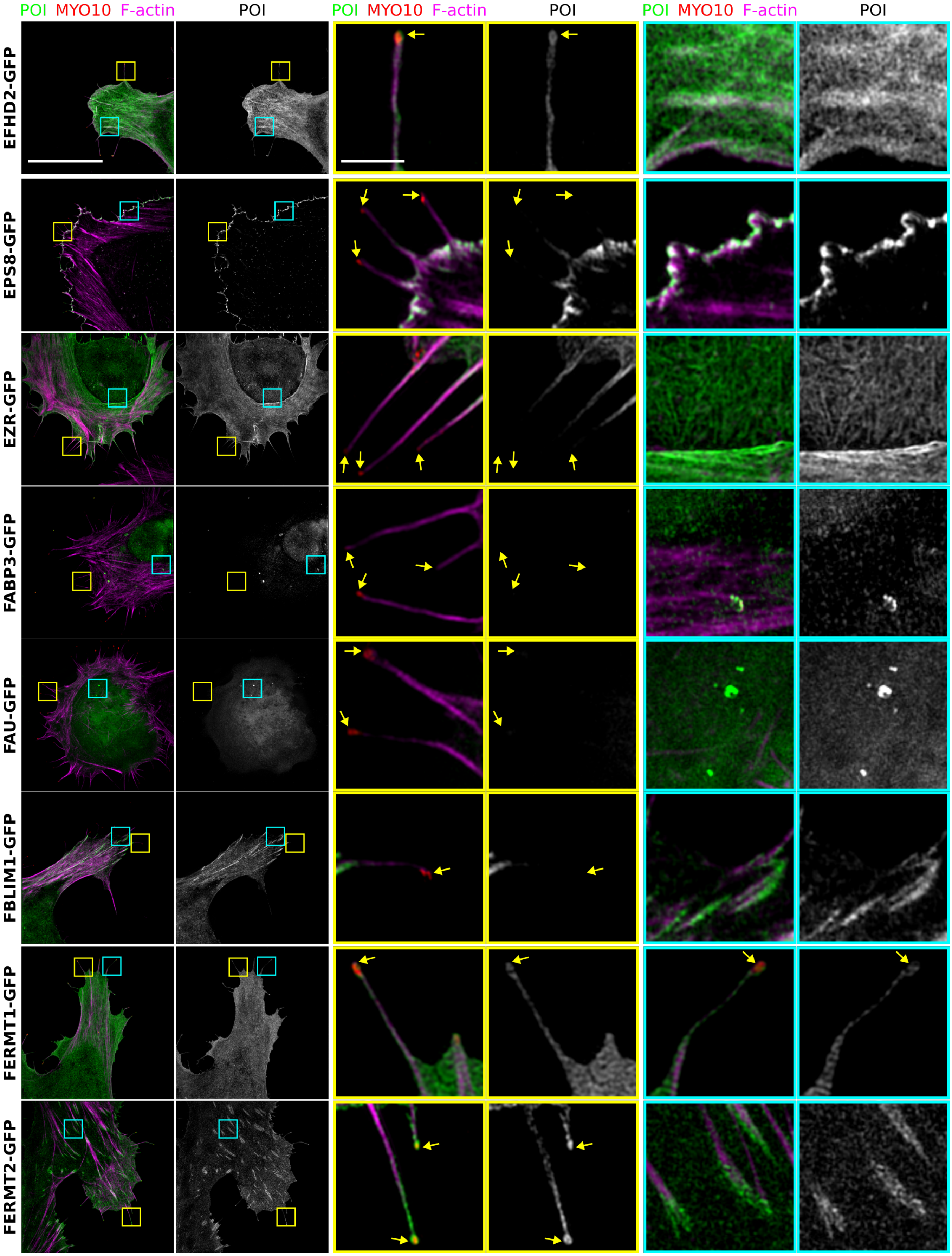

Representative images of each of the proteins imaged to generate the filopodia map. U2OS cells expressing GFP/RFP-tagged proteins of interest (POI, labelled with official human gene names) and GFP/RFP-MYO10 were plated on fibronectin for 2h, fixed and stained for F-actin before being imaged using SIM. A representative MIP is displayed for each condition. The blue and yellow squares highlight ROI, which are magnified; yellow arrows highlight filopodia tips; scale bars: (main) 20 µm; (inset) 2 µm.

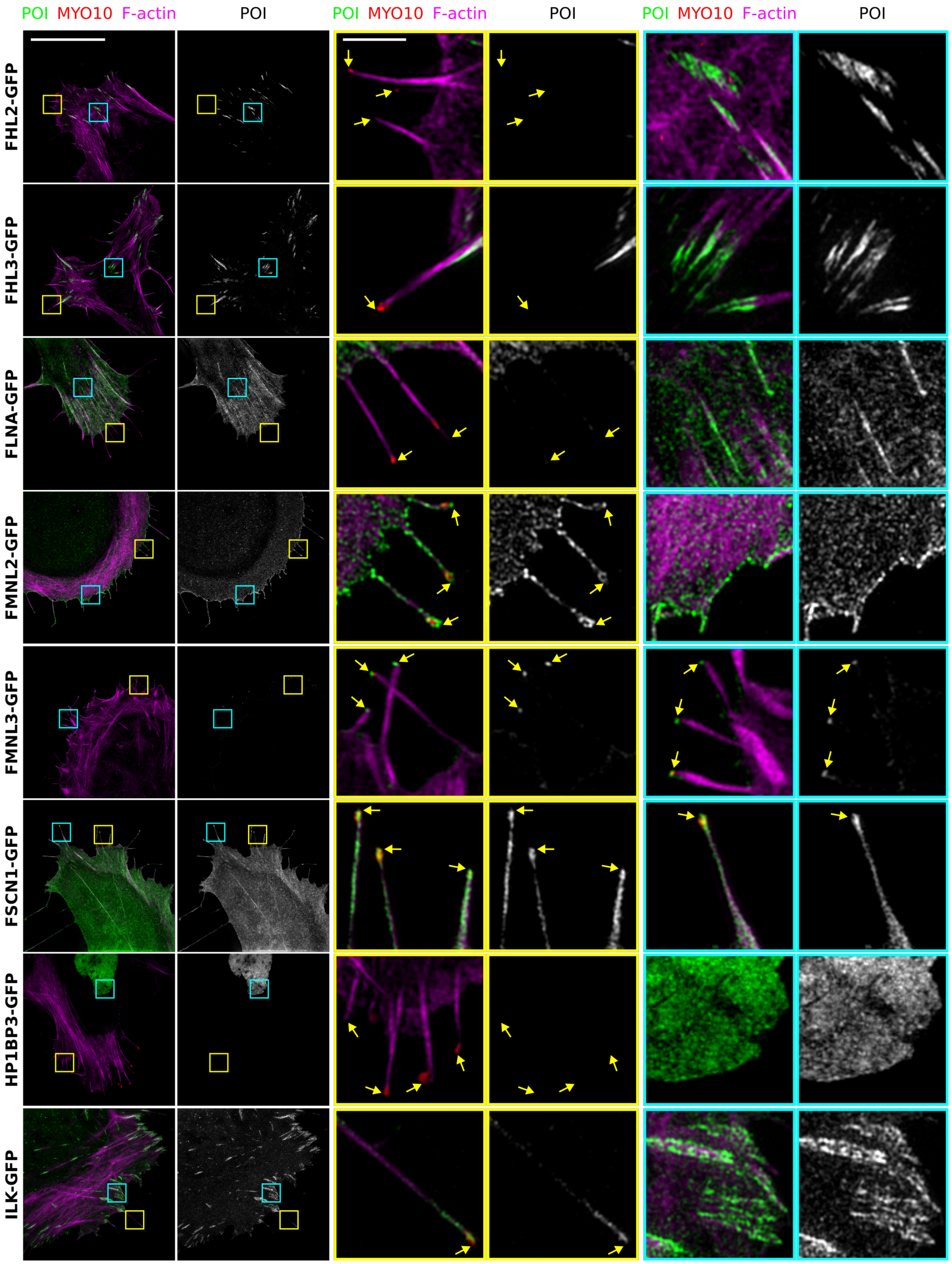

Representative images of each of the proteins imaged to generate the filopodia map. U2OS cells expressing GFP/RFP-tagged proteins of interest (POI, labelled with official human gene names) and GFP/RFP-MYO10 were plated on fibronectin for 2h, fixed and stained for F-actin before being imaged using SIM. A representative MIP is displayed for each condition. The blue and yellow squares highlight ROI, which are magnified; yellow arrows highlight filopodia tips; scale bars: (main) 20 µm; (inset) 2 µm.

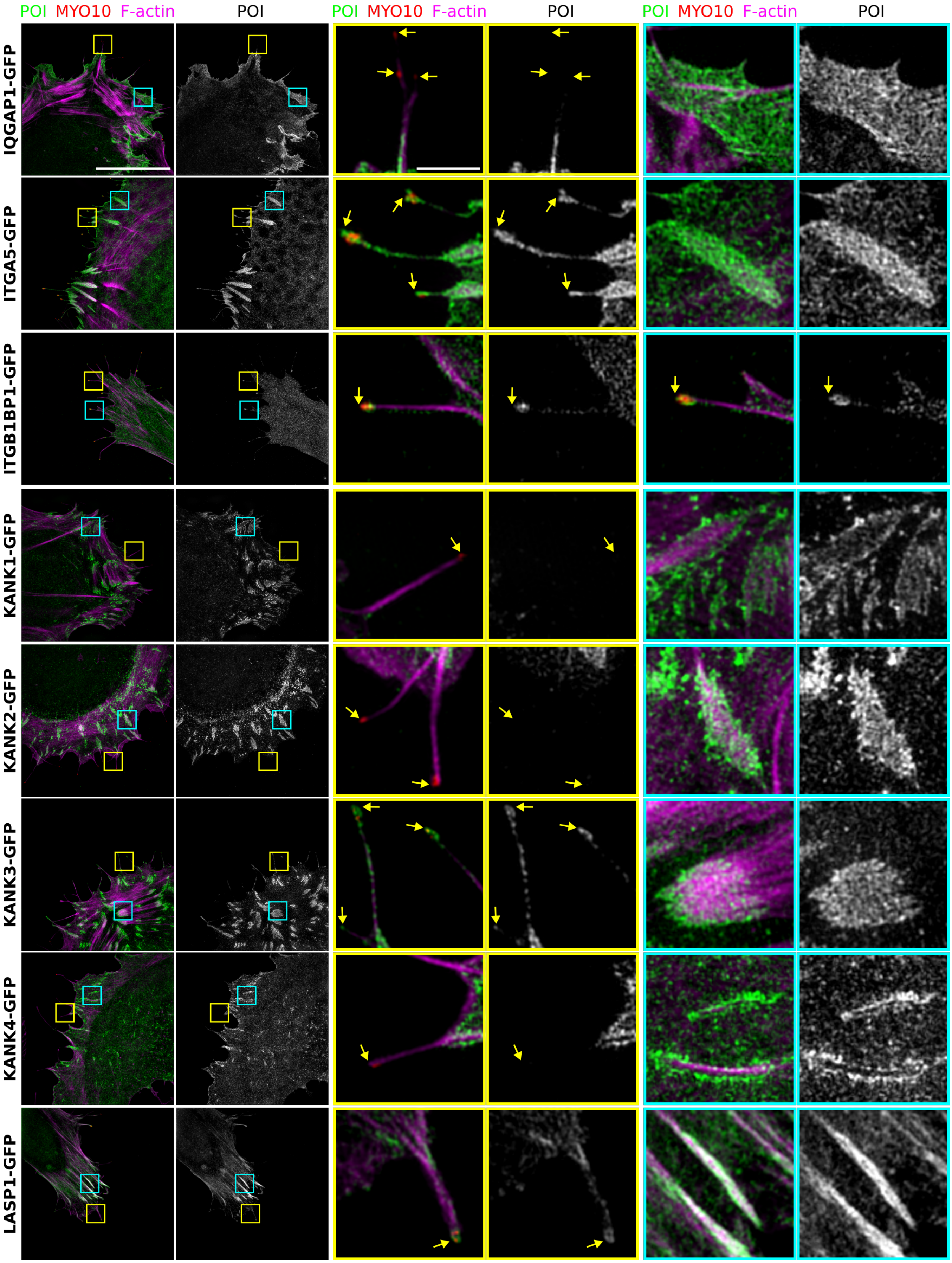

Representative images of each of the proteins imaged to generate the filopodia map. U2OS cells expressing GFP/RFP-tagged proteins of interest (POI, labelled with official human gene names) and GFP/RFP-MYO10 were plated on fibronectin for 2h, fixed and stained for F-actin before being imaged using SIM. A representative MIP is displayed for each condition. The blue and yellow squares highlight ROI, which are magnified; yellow arrows highlight filopodia tips; scale bars: (main) 20 µm; (inset) 2 µm.

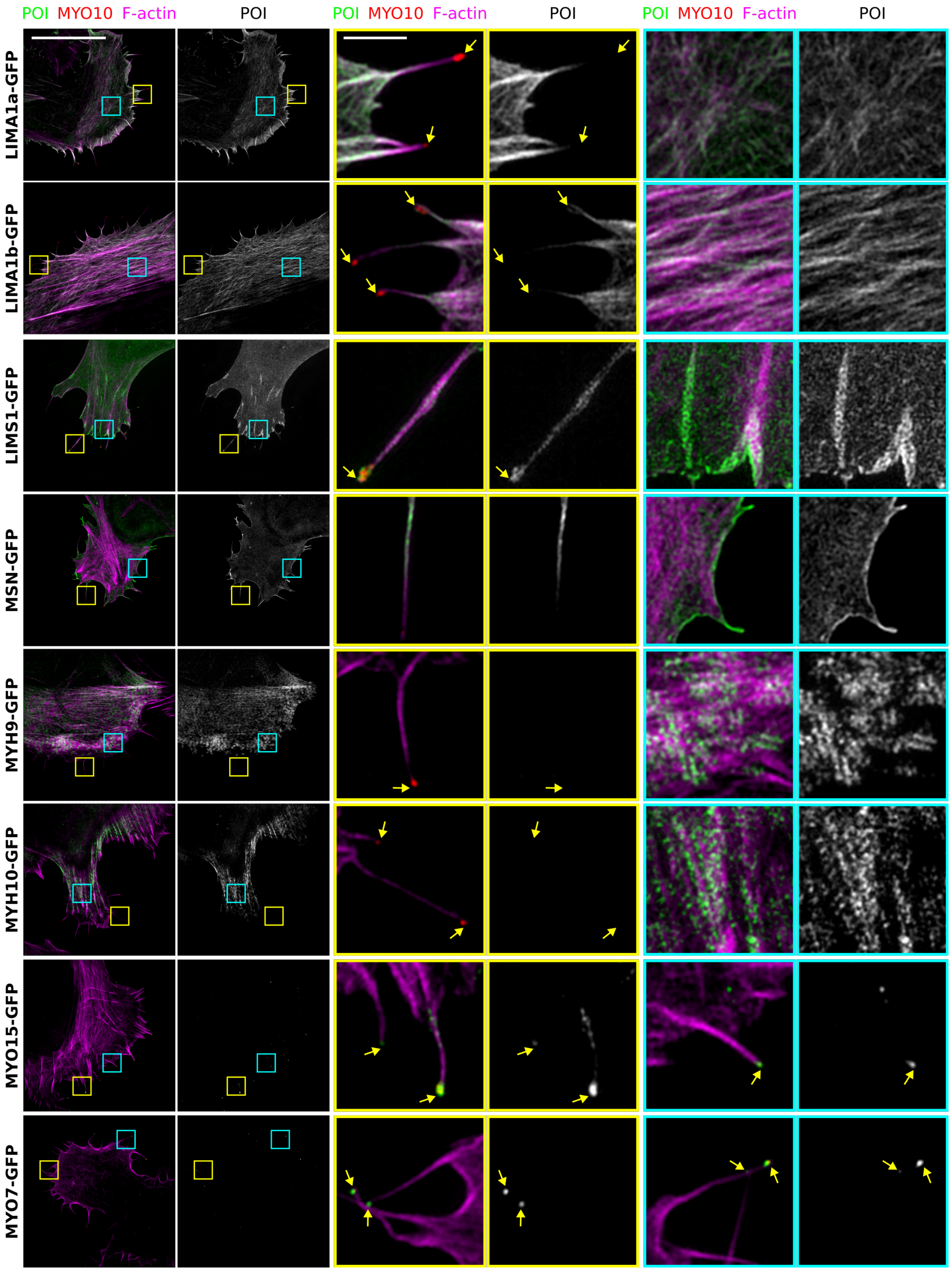

Representative images of each of the proteins imaged to generate the filopodia map. U2OS cells expressing GFP/RFP-tagged proteins of interest (POI, labelled with official human gene names) and GFP/RFP-MYO10 were plated on fibronectin for 2h, fixed and stained for F-actin before being imaged using SIM. A representative MIP is displayed for each condition. The blue and yellow squares highlight ROI, which are magnified; yellow arrows highlight filopodia tips; scale bars: (main) 20 µm; (inset) 2 µm.

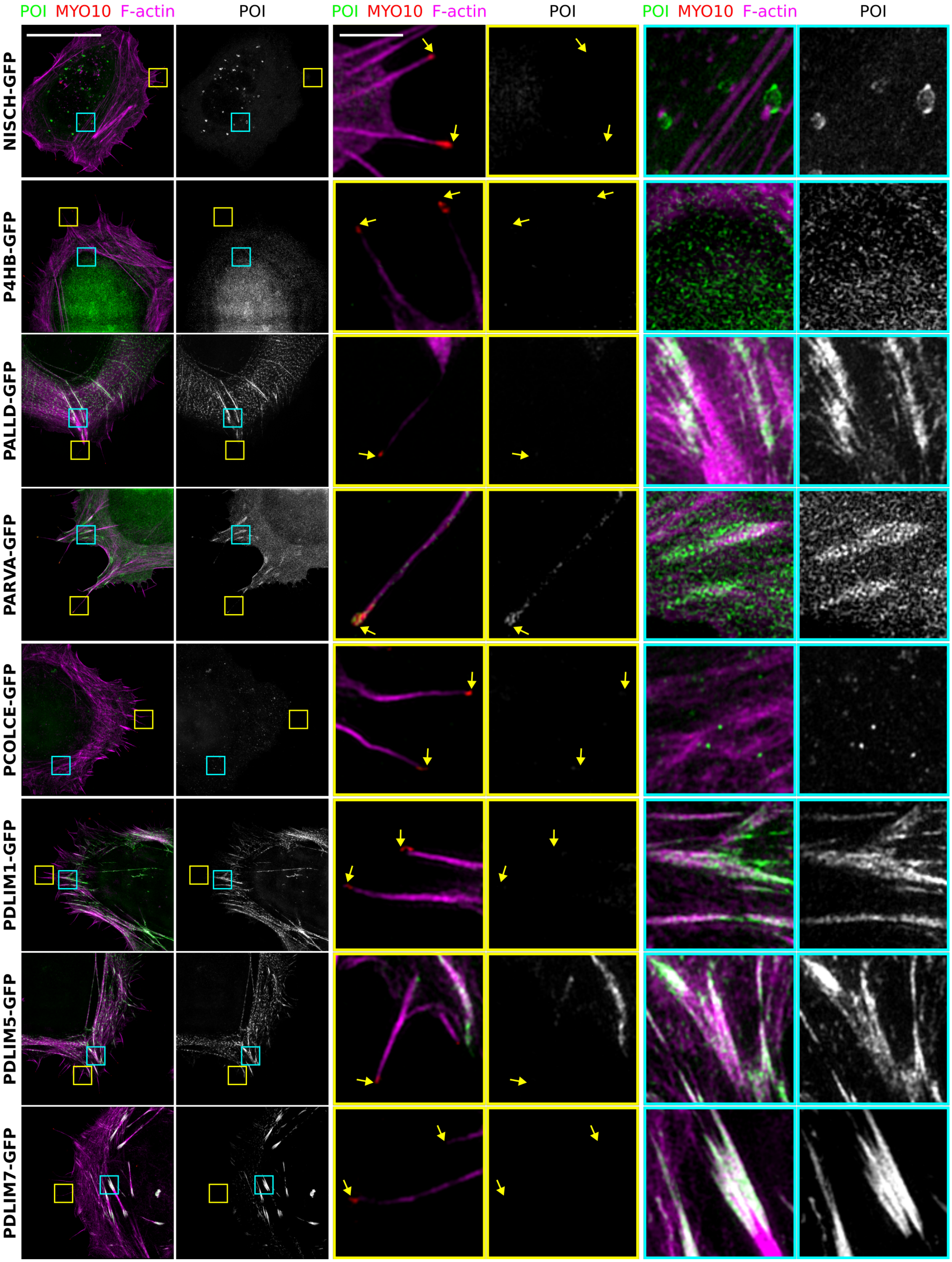

Representative images of each of the proteins imaged to generate the filopodia map. U2OS cells expressing GFP/RFP-tagged proteins of interest (POI, labelled with official human gene names) and GFP/RFP-MYO10 were plated on fibronectin for 2h, fixed and stained for F-actin before being imaged using SIM. A representative MIP is displayed for each condition. The blue and yellow squares highlight ROI, which are magnified; yellow arrows highlight filopodia tips; scale bars: (main) 20 µm; (inset) 2 µm.

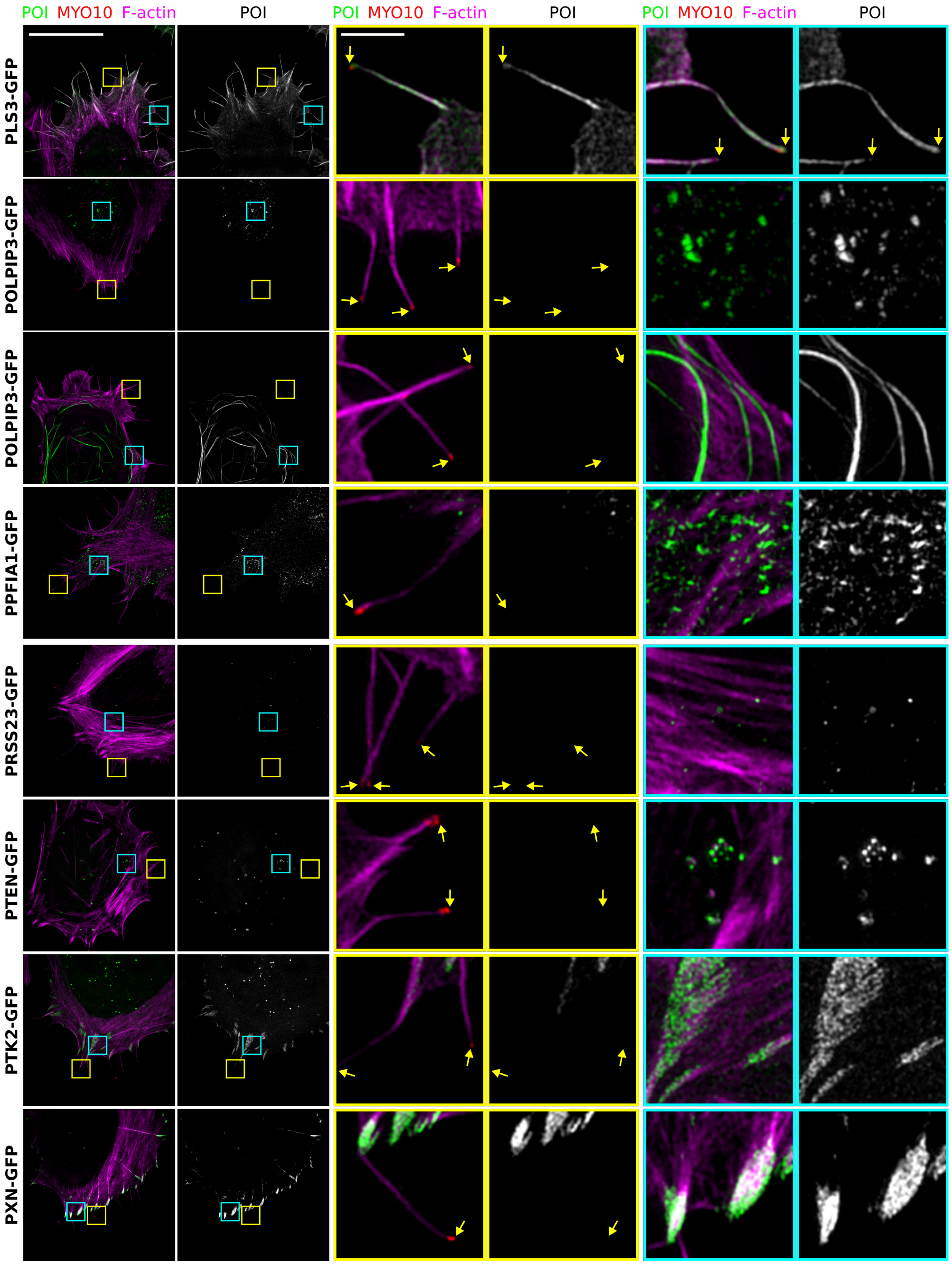

Representative images of each of the proteins imaged to generate the filopodia map. U2OS cells expressing GFP/RFP-tagged proteins of interest (POI, labelled with official human gene names) and GFP/RFP-MYO10 were plated on fibronectin for 2h, fixed and stained for F-actin before being imaged using SIM. A representative MIP is displayed for each condition. The blue and yellow squares highlight ROI, which are magnified; yellow arrows highlight filopodia tips; scale bars: (main) 20 µm; (inset) 2 µm.

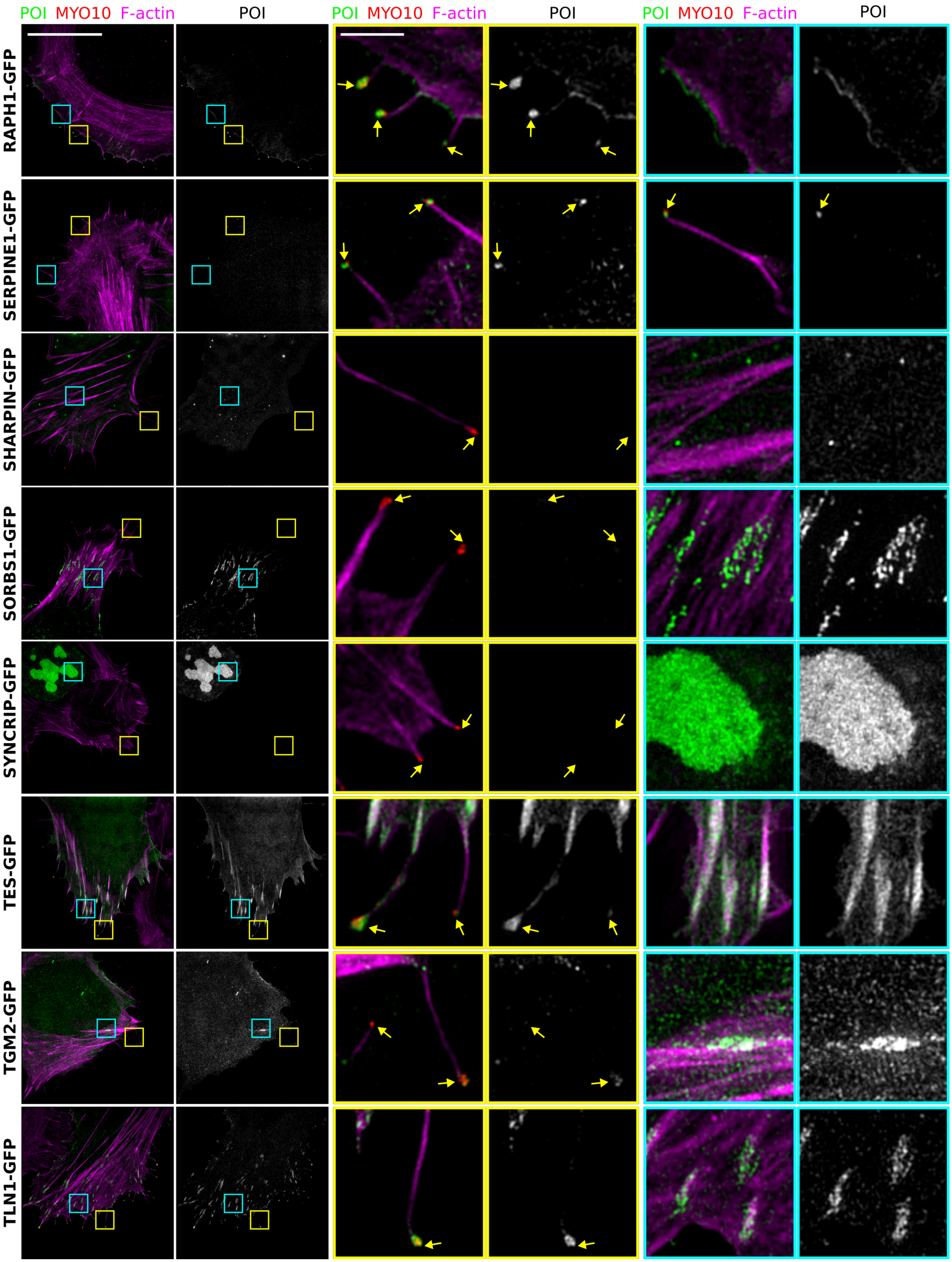

Representative images of each of the proteins imaged to generate the filopodia map. U2OS cells expressing GFP/RFP-tagged proteins of interest (POI, labelled with official human gene names) and GFP/RFP-MYO10 were plated on fibronectin for 2h, fixed and stained for F-actin before being imaged using SIM. A representative MIP is displayed for each condition. The blue and yellow squares highlight ROI, which are magnified; yellow arrows highlight filopodia tips; scale bars: (main) 20 µm; (inset) 2 µm.

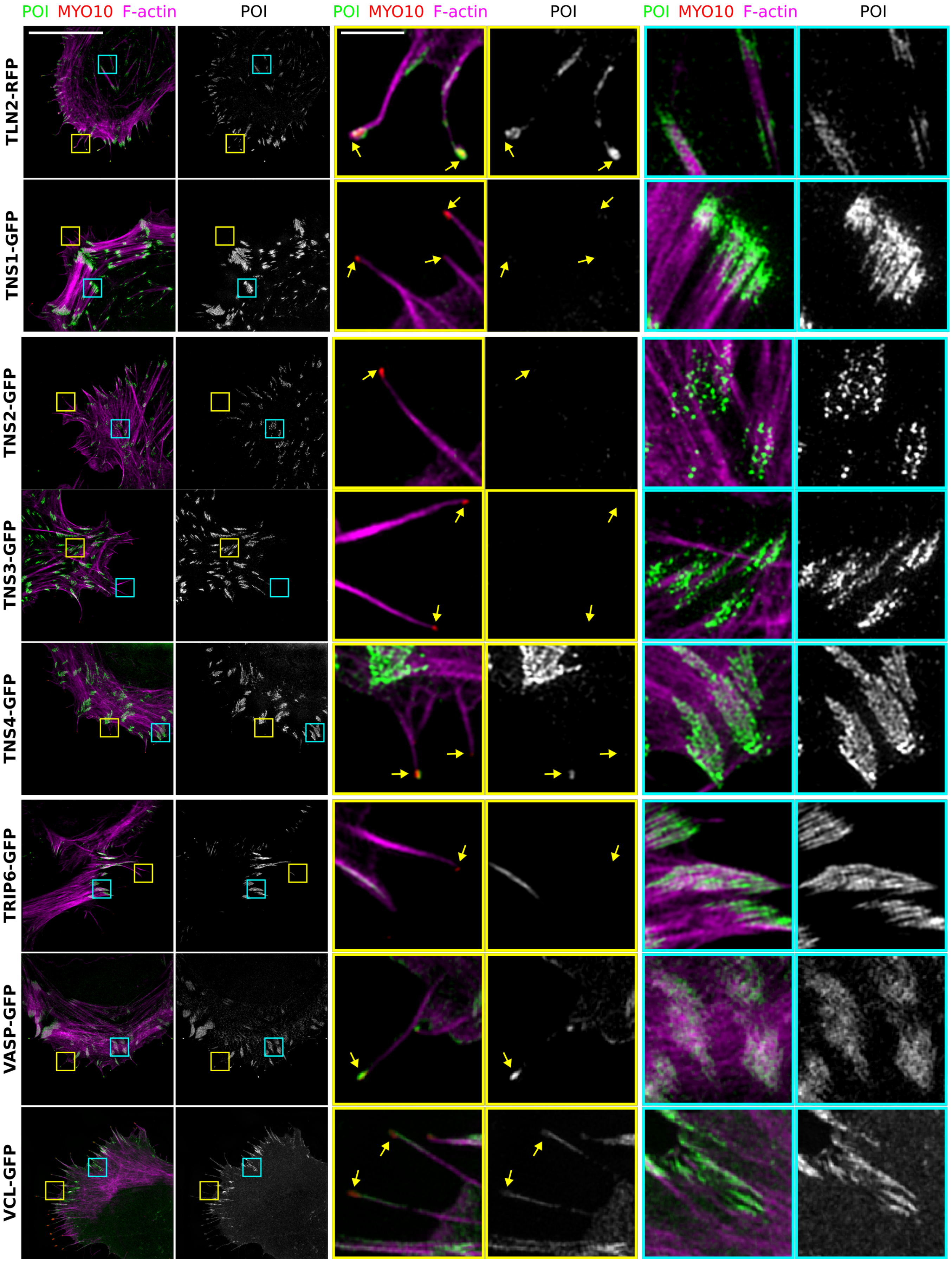

Representative images of each of the proteins imaged to generate the filopodia map. U2OS cells expressing GFP/RFP-tagged proteins of interest (POI, labelled with official human gene names) and GFP/RFP-MYO10 were plated on fibronectin for 2h, fixed and stained for F-actin before being imaged using SIM. A representative MIP is displayed for each condition. The blue and yellow squares highlight ROI, which are magnified; yellow arrows highlight filopodia tips; scale bars: (main) 20 µm; (inset) 2 µm.

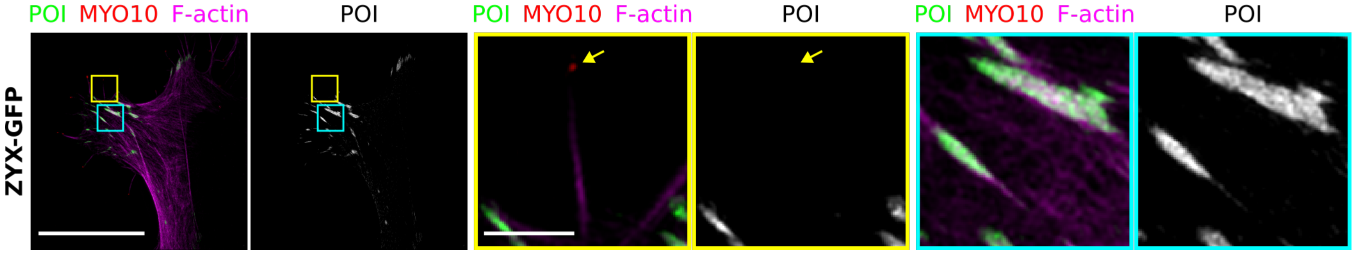

Representative image of zyxin-GFP in cells. U2OS cells expressing GFP/RFP-tagged proteins of interest (POI, labelled with official human gene names) and GFP/RFP-MYO10 were plated on fibronectin for 2h, fixed and stained for F-actin before being imaged using SIM. A representative MIP is displayed for each condition. The blue and yellow squares highlight ROI, which are magnified; yellow arrows highlight filopodia tips; scale bars: (main) 20 µm; (inset) 2 µm.

Sequence of the gene block used to clone mScarlet into pcDNA3.1-6His-MyoX

TTC GAC GGT ACC AAT GTT ACT AGT ATG GTG AGC AAG GGC GAG GCA GTG ATC AAG GAG TTC ATG CGG TTC AAG GTG CAC ATG GAG GGC TCC ATG AAC GGC CAC GAG TTC GAG ATC GAG GGC GAG GGC GAG GGC CGC CCC TAC GAG GGC ACC CAG ACC GCC AAG CTG AAG GTG ACC AAG GGT GGC CCC CTG CCC TTC TCC TGG GAC ATC CTG TCC CCT CAG TTC ATG TAC GGC TCC AGG GCC TTC ACC AAG CAC CCC GCC GAC ATC CCC GAC TAC TAT AAG CAG TCC TTC CCC GAG GGC TTC AAG TGG GAG CGC GTG ATG AAC TTC GAG GAC GGC GGC GCC GTG ACC GTG ACC CAG GAC ACC TCC CTG GAG GAC GGC ACC CTG ATC TAC AAG GTG AAG CTC CGC GGC ACC AAC TTC CCT CCT GAC GGC CCC GTA ATG CAG AAG AAG ACA ATG GGC TGG GAA GCG TCC ACC GAG CGG TTG TAC CCC GAG GAC GGC GTG CTG AAG GGC GAC ATT AAG ATG GCC CTG CGC CTG AAG GAC GGC GGC CGC TAC CTG GCG GAC TTC AAG ACC ACC TAC AAG GCC AAG AAG CCC GTG CAG ATG CCC GGC GCC TAC AAC GTC GAC CGC AAG TTG GAC ATC ACC TCC CAC AAC GAG GAC TAC ACC GTG GTG GAA CAG TAC GAA CGC TCC GAG GGC CGC CAC TCC ACC GGC GGC ATG GAC GAG CTG TAC AAG TCC GGACTC AGA TCC TAC CCC TAT GAT GTG CCT GAC TAT GCC GGA CAC GGA CAC GGC CAC CTG GTG CCC AGG GGC AGC AGA TCT CGA GCT CAAG CTT CGA ATT CTG CAG TCG ACG GTA CCA ATG TC

Sequence of the gene block used to clone BCAR1 CCHD into pEGFP-C1

CGT CGT CTC GAG GTT CTC GTC CAA TAG ACC ACG ACC TGG CCA ACT GGA CAC CAG CCC AGC CCC TGG TGC CGG GCC GGA CAG GGG GCC TGG GGC CTT CAG ACC GAC AGC TGC TGC TCT TCT ACT TGG AGC AGT GCG AGG CCA ACC TGA CCA CAC TGA CAG ATG CAG TGG ACG CCT TCT TCA CTG CGG TGG CCA CCA ACC AAC CAC CCA AGA TCT TTG TGG CAC ACA GCA AGT TTG TCA TTC TCA GTG CCC ACA AGC TTG TGT TCA TTG GGG ACA CAC TGT CAC GGC AGG CAA AGG CAG CTG ATG TCC GAA GCC AAG TGA CCC ACT ACA GCA ATC TGC TGT GTG ACC TCC TGC GTG GCA TTG TGG CCA CCA CCA AGG CTG CTG CCC TGC AGT ACC CAT CCC CTT CCG CTG CCC AGG ACA TGG TGG ACA GGG TCA AGG AGC TAG GCC ACA GCA CTC AGC AGT TCC GCC GCG TCC TGG GCC AGC TAG CTG CTG CCT GAG GAT CCC GTC GT
